# Graph-Based Machine Learning Identifies Oxygenated Block Polymer Replacements for Conventional Plastics and Elastics

**DOI:** 10.1021/jacs.5c21416

**Published:** 2026-03-04

**Authors:** Soheila Molaei, Kam C. Poon, Chang Gao, Katharina H. S. Eisenhardt, Matilde Concilio, Gregory S. Sulley, David Kohan Marzagão, Georgina L. Gregory, David A. Clifton, Clive R. Siviour, Charlotte K. Williams

**Affiliations:** † Department of Engineering Science, 6396 University of Oxford, Parks Road, Oxford OX1 3PJ, U.K.; ‡ Department of Chemistry, University of Oxford, Chemistry Research Laboratory, 12 Mansfield Road, Oxford OX1 3TA, U.K.; § Bush House, Strand Campus, 30 Aldwych, 4616 King’s College London, London WC2B 4BG, U.K.

## Abstract

Oxygenated block polymers, comprising esters and carbonates, are priority materials to replace petrochemical polymers in a circular plastics economy. These materials should repopulate the thermomechanical property space mapped by current plastics and elastomers. Here, a novel machine learning approach, PolyReco, predicts structures of oxygenated block polymers meeting the mechanical performance thresholds for widely used and hard-to-replace petroleum derived hydrocarbon polymers. Triblock oxygenated polymers are represented as graphs, and a link prediction algorithm enables feature extraction to identify new block polymer combinations, and associated degrees of polymerization, to meet the target properties. PolyReco is paired with a visualization tool for further material down selection based on user requirements. Three case studies highlight and experimentally validate its predictive power for identifying high-performance oxygenated block polymers, with new block polymers prepared and tested. These new block polymers exhibit tensile mechanical properties in the range of high-impact polystyrene, poly­(dimethylsiloxane), and styrenic elastomers; the experimental results indicate that PolyReco may help support the identification of sustainable materials that could reduce dependence on fossil-based polymer incumbents.

## Graphical abstract

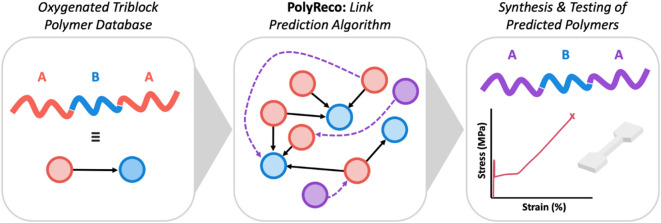

## Introduction

Our plastics and elastic production, use, and end-of-life options are unsustainable, resulting in both waste and carbon dioxide emissions crises.^1–3^ More sustainable polymers are urgently needed; to accelerate uptake, their properties must allow for the straightforward replacement of current fossil-derived materials.^4^ Oxygenated polymers, specifically polyesters and polycarbonates, are high-priority sustainable alternatives since they are readily derived from biomass or even directly from CO_2_-waste.^5–7^ Their manufacturing typically takes advantage of low energy and highly controlled heterocycle ring-opening polymerization routes.^8,9^ The resulting oxygenated linkages allow these materials to be both mechanically and chemically recycled.^10^ Oxygenated polymers usually show lower energy and greater selectivity for chemical recycling than conventional hydrocarbons.^8,11^ For some materials, biodegradation is also feasible and expected to be necessary where environmental dispersal is unavoidable.^12^

Currently, the leading commercial oxygenated polymers are homopolymers, e.g., poly­(l-lactide) (PLLA), poly­(caprolactone) (PCL) or poly­(propylene carbonate) (PPC).^10^ These products can populate some areas of the property space of petro-derived polymers, but matching properties of current high-performance engineering plastics and elastomers requires further innovation.^13^ Oxygenated block polymers have the potential to meet a broader material property range both for plastics and elastomers.^14^ Further, thermoplastic block polymers are important in the circular economy, since block phase separation optimizes mechanical properties while also enabling closed-loop mechanical recycling.^15^ This contrasts with chemically cross-linked thermosets, which typically cannot be recycled.^16^ Block polymer phase separation depends both upon the specific block chemistries and the degree of polymerization (DP) of each component block; identifying the right combinations is essential to deliver the best mechanical performances.^15,17^

Traditional polymer development targeting mechanical properties usually involves iterative synthesis and materials testing, which can be time-consuming and limit the exploration of property space.^18^ Recently, machine learning (ML) techniques have helped in many areas of polymer development and design by providing property predictions.^19–24^ Several excellent ML tools effectively predict polymer thermal properties, e.g., glass transition/melting temperatures, or conductivity.^25–30^ A few ML approaches also predict mechanical properties, e.g., stress and strain at break.^31–35^ Usually, ML results are compared against computational models; however, there is great power in experimentally validating predictions.^36,37^

One priority area for ML is in the prediction of block polymer structures and their mechanical properties.^38^ It is understood that block polymer phase-separated nanostructures lead to improved mechanical performance compared to the constituent homopolymers.^15^ ML techniques have even been harnessed to predict how to achieve block phase-separation.^39–44^ These advances highlight the potential of ML to not only guide the design of block copolymers with tailored nanostructures, but also to aid in the discovery of application-specific materials.

Almost all ML methods reported in the literature are trained and validated using established and commercial hydrocarbon-based homopolymers, this is because there are already widely available, large data sets for these materials.^20^ In the context of a future circular economy, these hydrocarbon polymers may not be such effective models for the properties or structures of oxygenated polymers.^2^ This presents a challenge, since there are far fewer commercial oxygenated homopolymers and a more limited range of available experimental data.^31,45–47^

The need to employ ML specifically to oxygenated polymers has only recently been recognized in the literature.^31,45,46,48,49^ In this field, ML has been used to study and predict physical properties, such as enthalpies of polymerization and biodegradability, which are unique to oxygenated polymers. For example, Ramprasad and co-workers reported the successful use of an ML model, based on a graph neural network (GNN) algorithm, to predict the ring-opening enthalpy, which is a fundamental thermodynamic parameter, essential to forward and depolymerization processes of oxygen-containing monomers.^47,50^ Olsen and co-workers demonstrated the use of an ML classification model to predict the biodegradability of polyesters.^45^ Further, ML has been used to help select replacement materials for currently used fossil-derived polymers.^45^ For example, to help guide the selection of biobased polymers as a replacement for current poly­(ethylene terephthalate) (PET) polymers, Beckham, Crowley, Broadbelt, and co-workers reported an ML model (PolyID), that allows for the prediction of thermal and gas transport properties of biobased polymers.^46^ The predicted properties for one of the biobased PET analogues, and thus its potential as a PET replacement, were experimentally verified. However, no mechanical properties were considered in that study.

In order to predict structure–property profiles for oxygenated block polymers that can effectively substitute conventional materials, it is necessary to not only map thermal properties, but instead to consider wide regions of tensile strength, elasticity, toughness, and stiffness. Indeed, some recent studies have begun to explore the prediction of mechanical properties for oxygenated polymers.^49,50^ Pilania and co-workers developed multitask deep neural network models trained on nearly 23,000 polymer chemistries to predict key thermal, mechanical, and gas transport properties across a space of 1.4 million candidates.^49^ Using this approach, they identified 14 PHA-based bioplastics as potential replacements for major petroleum-derived plastics, alongside possible synthetic routes for these sustainable alternatives. Recently, some of us developed PolyAGM, a graph-kernel-based machine learning algorithm that converts full polymer structures into numerical fingerprints to predict thermal and mechanical properties from molecular-level motifs.^31^ When it was applied to bioderived ABA-block copolymer thermoplastic elastomers, with ester and/or carbonate linkages, PolyAGM accurately predicted the material’s glass transition temperatures and tensile properties, and it identified key structural features influencing performance. A key limitation of most current ML models for the prediction of mechanical properties of oxygenated polymers remains the limited data availability, particularly for systems with complex or diverse structures. One approach is to develop ML tools that operate effectively using small data sets.

Most prior studies on the mechanical properties of oxygenated polymers focused on predicting specific target values.^20,31,50^ However, many applications call for properties that meet defined performance thresholds.^13^ For example, designing an elastomer capable of achieving higher values of stress or strain at break than the threshold value is a significant benefit since it will also tolerate lower stress/strain values – naturally, the converse is not true. Another important factor is that for current commercialized polymers, different polymer grades meet specific property criteria.^2^ Thus, replacing a currently commercialized polymer is unlikely to be achieved by setting a single target value for tensile mechanical performance. When setting appropriate tensile mechanical thresholds and benchmarking them against currently used materials, it is beneficial to identify lower limits to the materials’ ultimate tensile stress (σ) and strain (ε) at break. For instance, styrenic block polymers are among the largest volumes of thermoplastic elastomers used in transportation, electronics, and consumer products.^51^ In the future, we may need to replace styrenic elastomers due to challenges in recycling and high embedded greenhouse gas emissions.^51^ Sustainable block polymer replacements should exceed the performance of most styrenic elastomers and therefore meet lower thresholds of σ > 15 MPa and ε > 800%.^52^

Here, a polymer recommender system (PolyReco) identifies oxygenated block polymers meeting mechanical property thresholds based on a graph link prediction method. ABA-polymer structures are targeted; this terminology describes triblock structures where the “A” and “B” segments are polymers with thermal transitions above (A) and below (B) the operating temperature range of the material. The objective is to use ML to identify new block chemistries and appropriate polymer degrees of polymerization (DP), to match or exceed the mechanical property thresholds set by widely used commercial materials. The algorithm is trained using a custom-built database where most polyesters and carbonates were prepared using heterocycle ring-opening polymerization (ROP) or heterocycle/heteroallene ring-opening copolymerization (ROCOP) methods. These synthetic routes are significant, since both are highly controlled providing access to polymers with narrow DP range (dispersity), and with defined composition, molar mass, end-group, and side-group chemistries.^14^ Because the polymers are prepared in such a controlled manner, the key ML predictions can be more accurately experimentally correlated and verified.

We depart from conventional property-prediction by casting polymer discovery as threshold-conditioned link prediction. Instead of regressing absolute values for individual polymers, the objective is to learn the compatibilities between blocks (with specified DPs) and propose new links, novel block combinations not observed in training, that are likely to satisfy application-specific, distinct thresholds for stress and strain. The stress and strain thresholds used here are representative examples chosen to reflect common industrial performance windows; PolyReco is fully flexible and can be applied with any user-defined target values. This event-level framing (i.e., predicting whether a candidate meets a required threshold) mirrors real materials selection, meeting specifications rather than matching a number, exploiting relational structure to remain effective in data-sparse regimes, and turns machine learning from mere property estimation into actionable recommendations of synthesizable candidates.

## Results and Discussion

### Polymer Recommender System – PolyReco

A database describing the experimental mechanical data for 143 oxygenated ABA-block polymers was compiled (see Supporting Information). The database encompasses both different block polymer chemistries and ABA-polymers with fixed chemistry but varying block DP. In the database, each block polymer is identified by its constituent A and B-blocks and represented as a graph with two nodes and one edge, with the ABA pattern encoded by the polymer edge direction (Figure 1a). Each node comprises the A or B polymer represented as its SMILES string repeat unit, and its associated DP (Figure 1b).^53,54^ PolyReco was trained on the database containing mechanical data and chemical structures, with 10% of the data reserved for testing. Link prediction algorithms were employed to forecast new combinations of ABA-polymers and their respective DP ranges (Figure 1c). Although the training set comprises a modest 143 ABA polymers, PolyReco is formulated as a dual-head, threshold-conditioned classifier that jointly evaluates tensile strength and strain-at-break, such that increases in degree of polymerization alone are insufficient to guarantee a positive prediction. This multi-criteria framing reduces the risk of trivial shortcut learning and instead encourages the model to identify chemically meaningful block compatibilities, while necessarily positioning PolyReco as a data-efficient ranking and hypothesis-generation tool rather than a universal predictor. PolyReco was also provided with a database of 331 oxygenated homopolymers that had not previously been incorporated into block polymers. This allowed it to recommend a diverse array of structures not limited to known ABA-polymers. For blocks absent from the training set, PolyReco operates in an inductive “cold-start” mode, generating node features directly from SMILES-derived chemical representations and block DP, without using any prior block copolymer performance data. PolyReco is paired with a visualization tool which enables further down selection of the ABA-polymer output structures based on user-defined parameters, such as preferred polymer chemistries or service temperature window. Recommended block polymer structures meeting different targeted mechanical property thresholds were experimentally synthesized and characterized to validate the predictions (Figure 1d).

**Figure 1:**
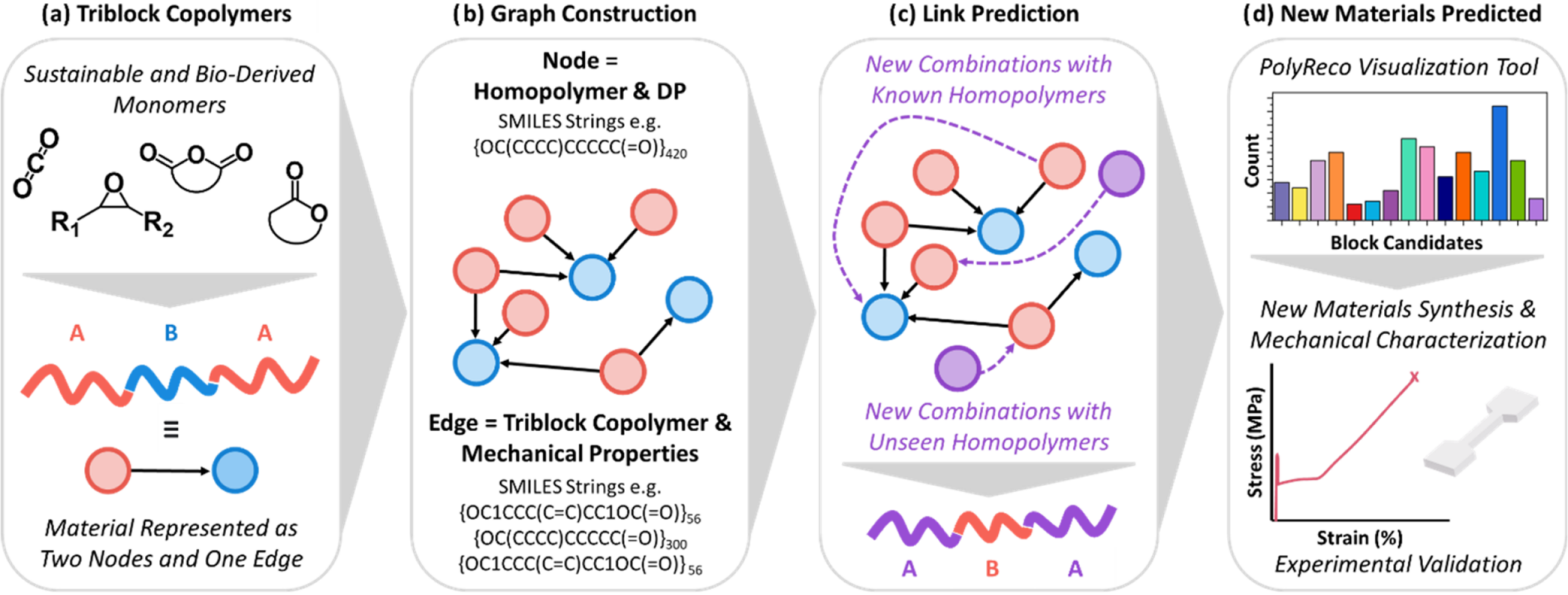
Overview of PolyReco. (a) Examples of oxygenated monomers used to synthesize the ABA-polymers. Each structure is represented as two nodes (red, blue) connected by directional arrows. (b) The repeat unit structure and DP are encapsulated within each node. (c) PolyReco predicts new block combinations (purple). (d) Down selection of recommended block candidates using the visualization tool, synthesis, and characterization of new block copolymers, and experimental validation of predicted properties.

### Link Prediction Algorithm

As previously mentioned, the PolyReco algorithm operates on a graph containing individual polymer blocks represented as nodes, with the edges that connect nodes representing the ABA-block polymers in the database and containing information about their mechanical properties. The algorithm is used to find new combinations of polymer blocks which meet desired mechanical property thresholds, i.e., form new edges in the graph between nodes that were not previously connected. In the field of graph neural networks, this is referred to as a link prediction task. More precisely, candidate ABA-block polymers are represented as a *directed block graph* G=(V,E) . Each node u∈V denotes an A-block defined by a monomer repeat unit (SMILES) and its degree of polymerization **DP**_***u***_ > **0**. A directed edge (u→v)∈E encodes block order for an ABA architecture; for symmetric ABA, the two outer A-blocks are merged by summing their DPs before adding a single edge ***A*** → ***B*** (the B-block DP is unchanged). All polymers in the data set follow this symmetric ABA architecture, and the two A-blocks are assumed to have identical DPs, so this mathematical merging step preserves the true underlying structure. Measured tensile properties (stress and strain at break (**τ**_**σ**_^**eng**^ **,τ**_**ε**_^**eng**^)) are stored as edge labels for supervised training.

There are three main steps in PolyReco’s link prediction process, as outlined in Figure 2. During the initialization step, each block’s molecular structure derived from its SMILES notation was defined as a feature vector, *F*_u1_, which contains information about the atoms and bonds in the block repeat unit (Figure 2a).

**Figure 2:**
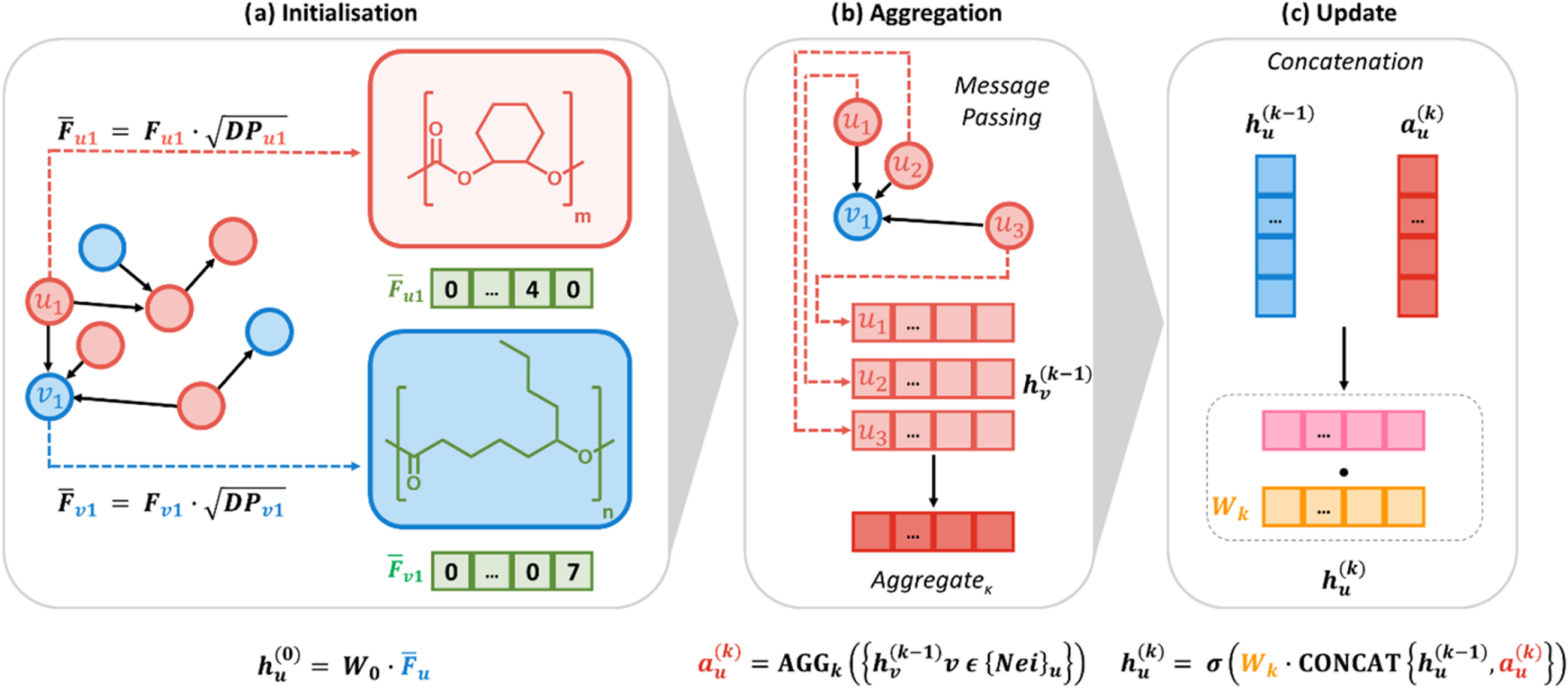
Link prediction stages for polymer prediction. (a) Nodes are initialized with feature vectors derived from SMILES notation. (b) Nodes aggregate information from their neighbors, leveraging local connectivity to enhance their feature vectors. (c) The aggregated information is used to update each node’s feature vector, incorporating the influence of surrounding structures.

More precisely, each block ***u*** is embedded as a fixed-length vector $F_{u}\in R^{d_{F}}$ that encodes the repeat unit graph (atom types and bonds), optionally enriched with physicochemical descriptors (logP, molar refractivity, topological polar surface area, H-bond acceptors/donors) and, when available, a SMILES language embedding using ChemBERTa (a learned sequence representation that complements the graph- and descriptor-based features).^55^

As the degree of polymerization, DP, is a determining parameter for polymers, we consider two monotone DP weightings: ${\overset{↼}{F}}_{u}=F_{u}\cdot \sqrt{DP_{u}}$ and

$${\overset{↼}{F}}_{u}$$

= ***F***_***u***_ · **log**(**max**(**DP**_***u***_ **,e**)). The square-root transform emphasizes the significance of longer blocks while tempering the influence of very large DPs; the logarithmic transform further compresses very long chains. This treatment acknowledges the nonlinear influence of polymer DP on properties, ensuring that longer chains are given more weight without allowing extremely long chains to overly dominate the feature weighting.^56^ The DP-weighted vector is then mapped to the encoder latent space by a learned linear projection $W_{0}\in R^{d\times d_{F}}$ , yielding the initial state ***h***_***u***_^**(0)**^ = ***W***_**0**_

$${\overset{↼}{F}}_{u}$$.

All feature coordinates are standardized (z-score) using training nodes only. Standardized in/out-degree cues may be concatenated to ***F***_***u***_ before the input projection; these are omitted from Figure 2 for clarity.

During aggregation, each node gathers structural information from its neighboring nodes through message passing, a core component of modern graph neural networks (Figure 2b).^57^ This step allows nodes to gather local structural information pertaining to how blocks might perform in combination with one another. Combining the features of neighboring nodes forms an aggregated vector. More precisely, at layer ***k*** ∈ {**1**,···,**K**}, node ***u*** aggregates information from its self-augmented neighborhood **Nei** ^+^(***u***) = **Nei**(***u***) ∪ {***u***} via a learnable, permutation-invariant operator **AGG**_***k***_ (Figure 2c)

$$a_{u}^{\left(k\right)}={\mathrm{AGG}}_{k}\left(\{h_{v}^{\left(k-1\right)}:v\in {\mathrm{Nei}}^{+}\left(u\right)\}\right)$$

In this instance, **AGG**_***k***_ is multihead graph attention with normalized coefficients over **Nei** ^+^(***u***); the equation remains valid for other neighborhood aggregators.

The aggregated vectors are then used to update each node’s feature vector in the update stage (Figure 2c). In the final link prediction step, the updated feature vectors of two nodes are combined and evaluated for potential link formation. This step uses a binary classification to identify whether a new block combination is expected to meet the mechanical property threshold set. More precisely, the aggregated vector is concatenated with the previous state and linearly projected; a pointwise nonlinearity is then applied (Figure 2c)

$$h_{u}^{\left(k\right)}=\phi \left(W_{k}\cdot \mathrm{CONCAT}\left[h_{u}^{\left(k-1\right)},a_{u}^{\left(k\right)}\right]\right)$$

with $W_{k}\in R^{d\times 2d}$ , $h_{u}^{\left(k\right)}\in R^{d}$ , and **ϕ** an elementwise activation (e.g., ELU). Residual connections, normalization, and dropout are applied after the projection (implementation detail, not depicted). After ***K*** layers, the encoder outputs $h\left(u\right)=h_{u}^{\left(K\right)}\in R^{d}$ . In code, we realize this update with multihead GAT and a residual connection (plus layer norm and dropout)

$$h_{u}^{\left(k\right)}=\phi \left(\mathrm{LN}\left({\mathrm{GAT}}_{k}\left(h^{\left(k-1\right)}\right)\right)\right)$$

which exemplifies the aggregate–then–update schematic in Figure 2c.

### PolyReco Accuracy for Mechanical Threshold Predictions

We evaluate the performance of PolyReco using several metrics, for which a brief overview is provided (Figure 3, further details in the Supporting Information, Figure S1). Accuracy alone, i.e., the proportion of correct decisions by the algorithm, often does not provide the full picture, as it does not distinguish false positives from false negatives. Precision (respectively recall), on the other hand, is the proportion of true positives over the sum of true positives and false positives (respectively false negatives). The F1 score is the (possibly weighted) harmonic mean between both precision and recall. AUROC quantifies the trade-off between true-positive and false-positive rates, whereas AUPRC integrates precision as a function of recall; both are termed “threshold-free” because they evaluate model ranking without fixing a decision threshold. Here, the macro versions of them are applied (precision, recall, etc.), which simply means the values are averaged over the two prediction tasks, i.e., stress at break σ and strain at break, ε. To mitigate overfitting in this data-sparse regime, PolyReco employs a deliberately regularized architecture, including dropout, L2 weight decay, gradient clipping, and validation-based checkpointing. For clarity, the engineering thresholds (**τ**_**σ**_^**eng**^, **τ**_**ε**_^**eng**^) that define the ground-truth labels are distinct from the probability thresholds used internally when computing operating-point metrics such as Precision, Recall, and ***F***_**1**_. Given the imbalance, AUPRC is the primary threshold-free metric; AUROC is reported for completeness.

**Figure 3:**
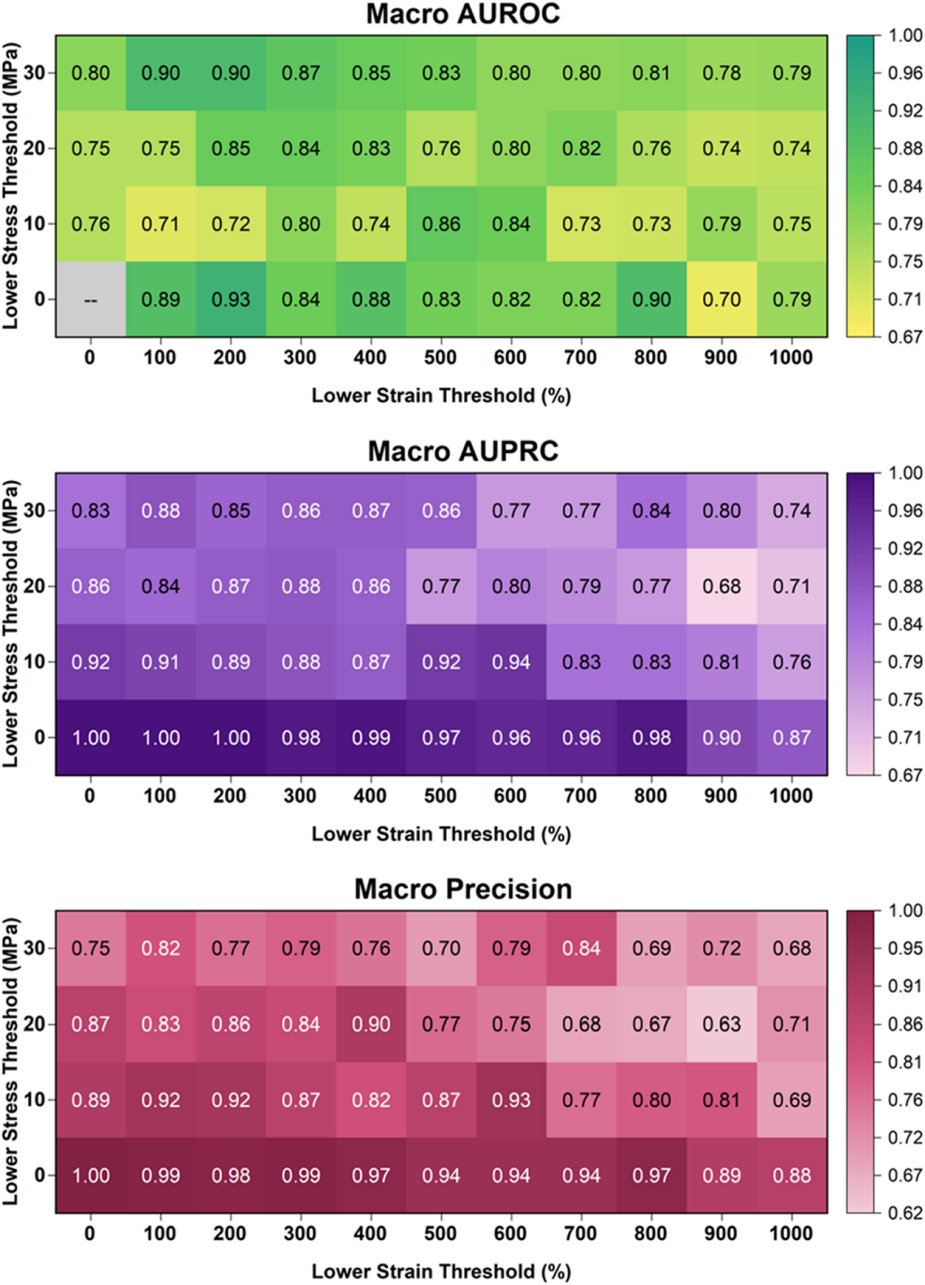
Performance assessment of PolyReco using log weighting (log­(DP)) across (τ_σ_^eng^, τ**_ε_^eng^**): macro-AUROC, macro-AUPRC, and macro-Precision (test, mean across folds).

All test-set metrics are computed on each fold of a stratified k-fold cross-validation, and summarized across folds as the mean ± standard deviation. Because positives, i.e., block combinations that satisfy the engineering stress and strain thresholds (**τ**_**σ**_^**eng**^, **τ**_**ε**_^**eng**^), are typically rare, plain accuracy is uninformative under class imbalance. Accordingly, the: (i) *threshold-free* ranking metrics, macro-AUROC and macro-AUPRC; and (ii) *operating-point* metrics, macro-Precision, Recall, and ***F***_**1**_ are reported (for mathematical definitions of these metrics, see the Supporting Information).

Across moderate engineering tensile mechanical threshold values, e.g., **τ**_**σ**_^**eng**^ = 10–20 MPa, **τ**_**ε**_^**eng**^ = 300–700%, log­(DP) weighting yields the strongest precision and competitive recall, leading to high macro- ***F***_**1**_ and macro-AUPRC (Figures 3, S1). For instance, at **τ**_**σ**_^**eng**^ = 10 MPa; and **τ**_**ε**_^**eng**^ = 600%, macro-***F***_**1**_ ≈ 0.85 with macro-AUPRC ≈ 0.94, precision remains high across **ε**-thresholds (often >0.90). This pattern indicates that **log**(**DP**) *saturates* the contribution of very long blocks and accentuates compositional signals, improving specificity when discriminating promising ABA-block polymer formulations from look-alikes that narrowly miss the criteria. When the stress target is stringent, e.g., **τ**_**σ**_^**eng**^ = 30 MPa, the square-root weighting (applied to DP) provides systematically higher recall and balanced macro-***F***_**1**_ (Figure S1). For example, at **τ**_**σ**_^**eng**^ = 30 MPa with **τ**_**ε**_^**eng**^ = 400–700%, macro-***F***_**1**_ ≈ 0.78–0.82, and macro-AUPRC stays >0.84, while recall improvements of +2–6% over **log**(**DP**) are observed. Because $\sqrt{\mathrm{DP}}$ grows sublinearly yet does not overly compress long-chain effects, it preserves a beneficial chain-length signal for the few, high-strength positives.

LP AUROC is uniformly high for both variants (validation 0.97–1.00, test 0.93–1.00; Figure S2), showing that the encoder learns chemically meaningful compatibilities between blocks, i.e., which A–B pairs occur. Thus, differences in classification arise primarily from how DP information is *scaled* in the block features rather than from a failure to recover connectivity. In practice, the log-DP weighting is best suited for precision-oriented down-selection at moderate performance targets, whereas the square-root DP weighting is preferable for exploratory searches at stringent strength thresholds, where maximizing recall of rare high-performance candidates is critical.

### Experimental Validation of Structure Predictions

Case studies focused on three different regions of materials property spaces were selected to assess the practical viability of PolyReco, with the objective of identifying block polymer structures that could meet the same mechanical property thresholds met by current petrochemical polymers. In evaluating the structures, PolyReco’s potential to predict combinations of polymers both present in the database but not previously reported within block polymers was examined. The aim throughout the work is not to identify exact, like-for-like material replacements, but to pinpoint candidates with comparable tensile mechanical profiles. These are intended as experimentally viable leads rather than drop-in substitutes, and comprehensive validation across additional properties remains an essential future step. This approach provides options for experimentally validating the model, testing whether the predicted mechanical behaviors align with the experimental results.

For each predefined σ and ε threshold value, PolyReco generated output ABA-polymer structures and the individual block DPs (Figure 4b). These output files were then evaluated using a visualization tool, which enabled a further down-selection process based on other critical parameters (Figure 4c, see Supporting Information). Namely, filters are applied to the output structures for block polymer chemistry, synthetic accessibility, and upper and lower *T*_g_ values, which may help define the processing and service temperature windows. For example, after selecting a specific B-block chemistry and filtering by desired thermal properties, the recommended A-block structures can be displayed. A range of DP for each block is then recommended for the specified A and B chemical structures. These outputs provide the comparable wt % of the A-block, calculated from the A to B-block ratio (see Supporting Information). For instance, to target thresholds representative of elastomeric property space, block ratios were consistently below <30 wt % A-block, aligning with typical block polymer phase diagrams.^14^ The PolyReco visualization tool enabled straightforward selection of an ABA-polymer for synthesis from the generated output structures predicted to meet the predefined σ and ε thresholds for a targeted mechanical property space. The selected polymer structure was subsequently synthesized for experimental validation (Figure 4e). All the new materials were produced using one-pot polymerization methods, utilizing controlled polymerizations, namely ROP of cyclic monomers and anhydride/epoxide and carbon dioxide/epoxide ROCOP.^14^ These polymerization types allow for good control over A and B-block DP, with a narrow dispersity.

**Figure 4:**
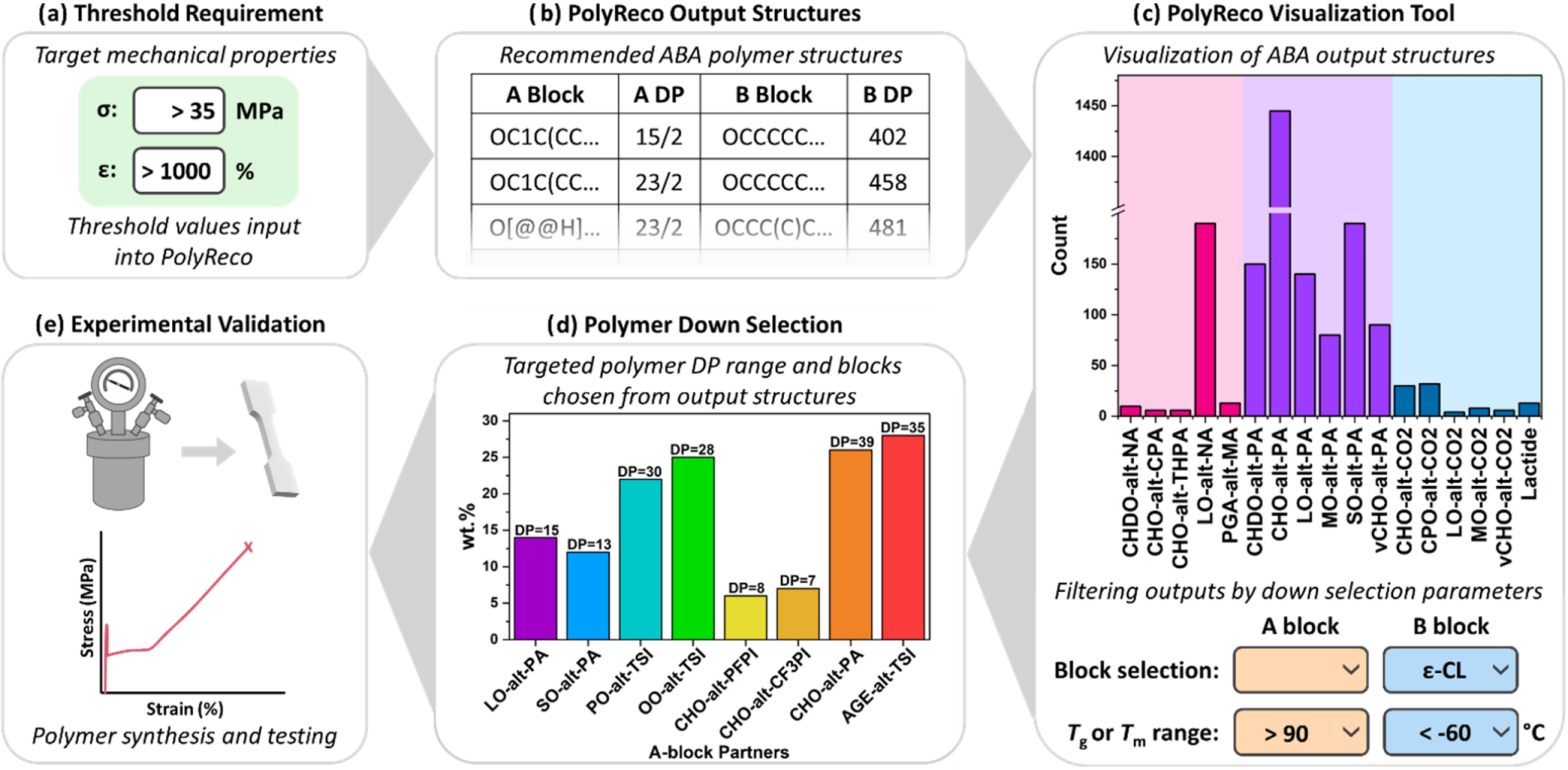
Example workflow for using PolyReco to predict polymer structures for Case Study 1. (a) Defining PolyReco input threshold values. (b) Obtaining PolyReco structural outputs. (c) Visualization and (d) Down selection of polymer structures based on desired block chemistries, thermal properties, and synthetic accessibility. (e) Polymer synthesis and experimental validation by mechanical characterization.

### Case Study 1: High-Impact Polystyrene

High-impact polystyrene (HIPS) is integral to various industries, such as packaging and consumer electronics, due to its exceptional mechanical properties, particularly σ and ε.^58^ However, HIPS presents significant environmental challenges: its structure features permanent chemical cross-links and thus is not easily recyclable, it contributes to environmental pollution, and it has significant embedded carbon dioxide emissions (2.6 kg CO_2_ equiv./kg polymer).^59^ In the search for recyclable alternatives to HIPS, PolyReco was tasked with predicting ABA-polymer structures with σ > 35 MPa and ε > 1000%, resulting in ∼ 14,000 recommended output structures.

Poly­(caprolactone) (PCL) was selected as a component for down selection as the B-block owing to its commercial production and potential for both mechanical and chemical recycling.^60,61^ From the refined output structures, poly­(cyclohexene oxide-*alt-*phthalate) (Poly­(CHO-*alt*-PA)) was then selected for the A-blocks as it has *T*_g_ values above 100 °C, which are comparable to the 90 °C for HIPS (Figure 5).^58,62,63^ For this combination of A and B-blocks, predicted DPs of B ≈ 500 and A ≈ 40 were selected (see Supporting Information). Experimentally, the ROP of CL monomer, along with the alternating ROCOP of PA and CHO, resulted in an all-polyester material featuring a PCL DP of 489 and poly­(CHO-*alt*-PA) total DP of 84 (detailed synthesis and characterization methods, see Supporting Information). The new block polyester thermoplastic was optically transparent, showed *T*_g_ values of −62 and 111 °C, and exhibited σ of 46.2 ± 6.4 MPa and ε of 1156 ± 101%. As a result, the new triblock polyester suggested by PolyReco displays mechanical properties that meet the thresholds set for HIPS properties, marking it as a promising oxygenated plastic alternative to HIPS, based on these factors (Table S2). Further, the combination of PCL and high *T*_g_ P­(CHO-*alt*-PA) is a novel block combination predicted by PolyReco.

**Figure 5:**
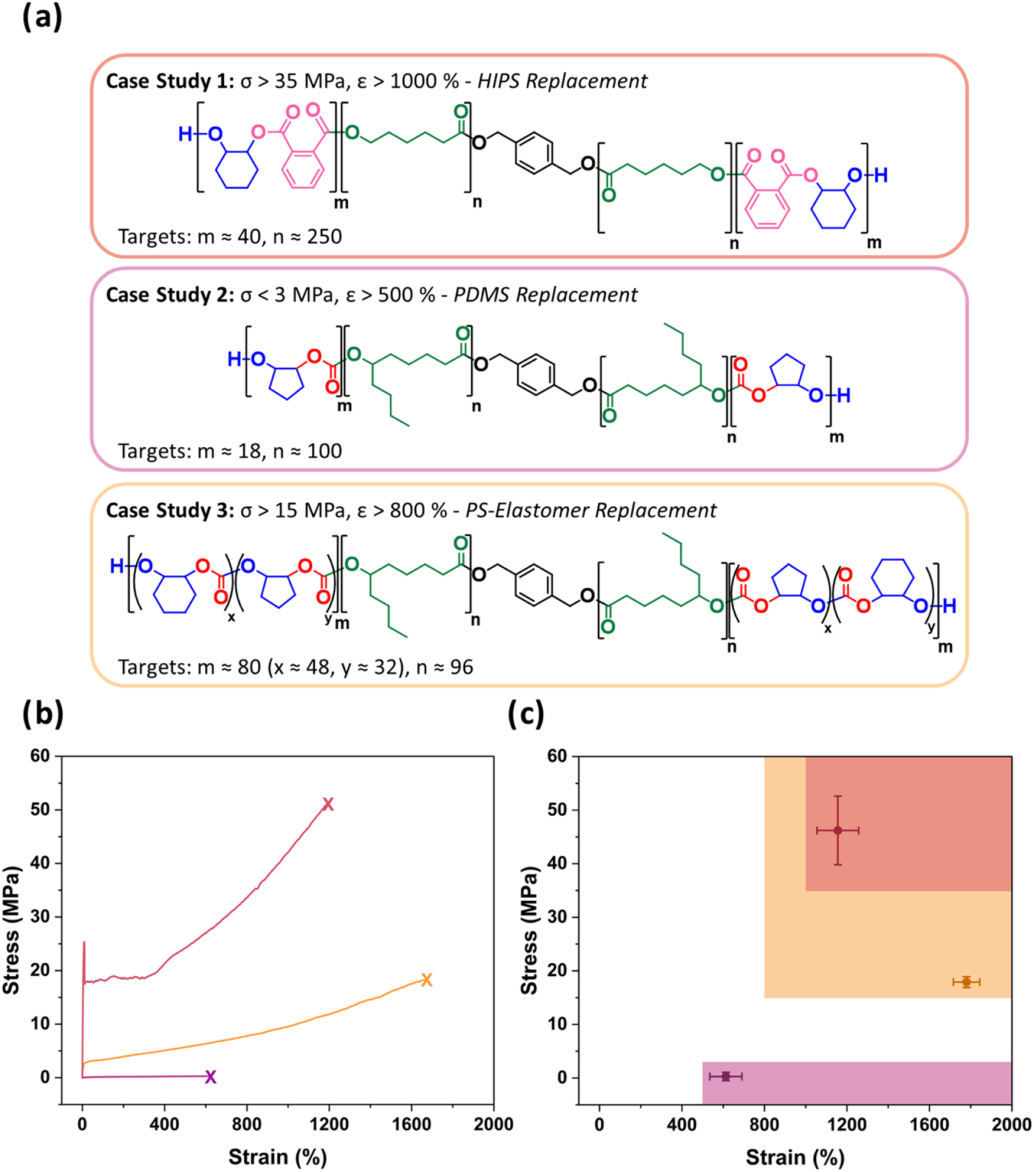
Experimental mechanical properties of new materials and comparison to predicted thresholds. (a) ABA structures and approximate DP values recommended by PolyReco for each case study. (b) Representative stress–strain curves for each case study material (10 mm min^–1^). (c) Stress and strain values at break for each case study material relative to targeted mechanical property thresholds (shaded areas). Error bars represent the experiment standard deviation for *N* = 5.

This case study illustrates PolyReco’s capacity to predict new materials in a targeted mechanical performance range using data from known homopolymer structures. It is also clear that other material parameters are important to plastic performance, and so the new block polyester’s toughness and Young’s modulus (*E*_y_) were also measured as 311 ± 61 MJ m^–3^ and 0.52 ± 0.06 GPa, respectively. The toughness compares well to HIPS, but *E*_y_ is lower. In future, a complete assessment of other properties relative to HIPS would require a broad range of impact testing (e.g., Charpy or Izod tests) and compressive mechanical evaluations. Thus, the limited data reporting against all these metrics is one of the limitations of the current study and future machine learning databases and tools should aim to accommodate additional variables and properties.

### Case Study 2: Polysiloxane

Polydimethylsiloxane (PDMS) is a versatile material widely used due to its excellent mechanical properties, including high elasticity, low glass transition temperature, and significant strain at break.^64^ These attributes make PDMS ideal for use in microfluidics, flexible electronics, and biomedical devices, where its flexibility is crucial. However, PDMS presents significant challenges from a life cycle assessment perspective. Its production is energy-intensive and results in embedded greenhouse gas emissions exceeding 6 kg CO_2_ equiv/kg of polymer.^65^ Additionally, PDMS is difficult to recycle, and cross-linking PDMS chains further complicates its environmental impact.^66^ In order to reproduce the performance of PDMS using oxygenated polymers and to evaluate PolyReco in a very different region of the mechanical property space, a target threshold of σ < 3 MPa and ε > 500% was set. This case study also allowed investigation of PolyReco’s performance in predicting new block combinations using block structures that were not part of the training set.

Poly­(cyclopentene carbonate) (PCPC) was selected as an A-block candidate that was not present in the training set. Recent reports have shown PCPC has promising homopolymer properties due to its low entanglement molecular weight and high tensile strength.^67^ It can be synthesized from cyclopentene oxide (CPO) and utilizes CO_2_ as an abundant renewable resource. The predicted structures for the PDMS threshold region were filtered for those containing PCPC blocks and DP ≈ 18 was targeted. ABA structures with poly­(ε-decalactone) (PDL) B-blocks (DP ≈ 200) were selected for synthesis as they fell within the target range, and PDL can be bioderived. A PCPC-PDL-PCPC triblock polymer was successfully synthesized with block DPs of 32-196-32. It gave σ and ε values within the target thresholds at 0.34 ± 0.08 MPa and 613 ± 78%, respectively (see Figure 5b,c). These results highlight the algorithm’s ability to predict materials that meet low mechanical property threshold requirements, one of which is an upper bound. Additionally, they demonstrate the capability to identify new materials with previously unseen block combinations. This underlines PolyReco’s potential to integrate new homopolymer structures into the database, as well as for the accelerated discovery of innovative block polymer structures. One limitation is that the novel ABA-polymer exhibits a higher *T*_g_ (−51 °C) than PDMS (*T*_g_ = −123 °C), however, it exhibits the lowest *T*_g_ of the 4248 structures predicted to meet the set mechanical thresholds. This highlights a knowledge-gap in the field for very low *T*_g_ oxygenated polymers.

### Case Study 3: Styrenic Block Polymer Elastomers

Styrenic block copolymers (SBCs) are widely used in the automotive and construction industries.^68^ These elastomers are tough and able to withstand significant strain, benefiting uses as adhesives, sealants, and flexible packaging.^51^ However, they are derived from petroleum-based resources and comprised of non-degradable linkages, raising long-term sustainability concerns regarding their production and disposal.^69^

Styrenic-based elastomers require mechanical properties meeting the specification of σ > 15 MPa and ε > 800%.^68^ Here, PolyReco was tasked with predicting structures containing more sophisticated terpolymers within the blocks. Recent reports have shown that the polycarbonate synthesized from a mixture of CPO, CHO, and CO_2_ can deliver toughened plastics.^70^ PolyReco predicted that poly­(CPO-*co*-CHO-*alt-*CO_2_) (PCPC-*co*-PCHC) might be a good A-block candidate (DP_PCHC_ ≈ 48 and DP_PCPC_ ≈ 32) to replace the polystyrene segment in SBCs, and in combination with a PDL B-block (DP_PDL_ ≈ 182) could give promising new tough elastomers. The synthesized material has a central PDL block DP of 187, while the outer polycarbonate block is composed of DP_PCHC_ = 48 and DP_PCPC_ = 30 (Figure S14). Tensile testing determined σ = 17.9 ± 1.0 MPa and ε = 1780 ± 64% (Figure 5b), exceeding both the property thresholds (Figure 5c). The mechanical performance is comparable to that of styrenic-based elastomers, along with the thermal properties (Figure S17). This case study illustrates that the model can predict more complex terpolymer block chemistries that were not present in the training database.

It is important to emphasize that, although the synthesized materials demonstrate mechanical performance comparable to that of petrochemical incumbents, a comprehensive life cycle assessment (LCA) and detailed techno-economic analyses would certainly be required to verify whether these materials indeed offer a more sustainable and cost-effective alternative. Such process and sustainability engineering evaluations are clearly well beyond the scope of identifying candidate polymer chemistries for research exploration. It would be interesting, in future, to consider how to integrate valid sustainability and techno-economic data into the visualization tool or into the material down-selection parameters.

## Conclusion

Graph-based machine learning utilizing link prediction methods was applied to accurately predict new ABA-block polymer structures meeting target tensile mechanical property thresholds. The algorithm, PolyReco was coupled with a visualization tool to identify polymer structures and enable down-selection by other key parameters, such as thermal properties or synthetic accessibility. The potential of PolyReco was experimentally validated through three case studies, which were used to predict oxygenated block polymer replacements for high-impact poly­(styrene), poly­(dimethylsiloxane), and styrenic block copolymer elastomers. The predicted block polymer chemistries illustrate how PolyReco can identify new combinations of polymers. PolyReco was also able to predict structures from polymers that were not previously reported in block architectures, including more sophisticated terpolymers. In all three examples, the experimental mechanical properties of the predicted materials matched the desired thresholds and, in several cases, the thermal properties were also a good match for existing limits. This research highlights the possibility of using machine learning methods to streamline sustainable polymer materials discovery. Future developments should build upon the database and platform to incorporate additional material properties, such as Young’s modulus, toughness, creep, or viscoelastic response, using the same learned polymer representations and training separate predictors for each descriptor, provided sufficiently curated data sets become available. PolyReco has the potential to significantly expedite sustainable polymer materials discovery, to help direct experiments to identify new combinations of ABA-block polymers to replace currently used plastics and elastomers, and to incorporate and update to include new polymer structures and chemistries as the field evolves.

## Supplementary Material



## Notes

The data-sets underpinning the computational and experimental results in the paper are available open-access at the following sites: PolyReco: https://github.com/SoheilaMolaei/PolyReco Visualization tool: https://github.com/GeorgeLGregory/PolyRECO-Visualization-Tool. Exerpimental Dataset: 10.5287/ora8nyk4opoz. The Supporting Information is available free of charge at https://pubs.acs.org/doi/10.1021/jacs.5c21416.

Detailed synthetic and characterization (NMR, SEC, DSC, TGA) procedures, instrument details, and ISO standards used for materials testing (PDF)
