# Supplementary material for: Graph-Based Machine Learning Identifies Oxygenated Block Polymer Replacements for Conventional Plastics and Elastics
Source: J Am Chem Soc. 2026 Mar 4;148(10):10934–44. doi: 10.1021/jacs.5c21416 (PMC13003491; doi:10.1021/jacs.5c21416)
Supplement: Supplementary file 1 [file ja5c21416_si_001.pdf]

# Supporting Information

## Graph-Based Machine Learning Identifies Oxygenated Block Polymer Replacements for Conventional Plastics and Elastics.

Soheila Molaei‡[a], Kam C. Poon‡[b], Chang Gao[b], Katharina H. S. Eisenhardt[b], Matilde Concilio[b], Gregory S. Sulley[b], David Kohan Marzagão*[c], Georgina L. Gregory*[b], David A. Clifton*[a], Clive R. Siviour*[d], and Charlotte K. Williams*[b].

[a] Institute of Biomedical Engineering, Old Road Campus Research Building, University of Oxford, Oxford, OX3 7DQ, UK

[b] Chemistry Research Laboratory, Department of Chemistry, University of Oxford, Oxford, OX1 3TA, UK

[c] Bush House, Strand campus, 30 Aldwych, King's College London, London, WC2B 4BG, UK

[d] Department of Engineering Science, University of Oxford, Parks Road, Oxford, OX1 3PJ, UK

The data-sets underpinning the computational and experimental results in the paper are available open-access at the following sites:

PolyReco: https://github.com/SoheilaMolaei/PolyReco

Visualization tool: https://github.com/GeorgeLGregory/PolyRECO-Visualization-Tool.

Exerpimental Dataset: 10.5287/ora-8nyk4opoz.

**Table of Contents**

Additional Computational Details....4

Model Description and Feature Construction....4

Two-Stage Learning....4

Stage 1: Link-prediction pretraining....4

Stage 2: Threshold-conditioned classifiers for stress and strain....5

Graph Neural Network Processing....5

Evaluation....5

Metrics....6

Labels and scores....6

Threshold-free metrics....6

Operating-point metrics....6

Results: Effect of chain–length weighting....7

Figure S1. a) Log weighting (log(DP)) across macro-recall, and macro-$F1$. b) Square-root weighting $DP$ across ($\tau\sigma eng, \tau\varepsilon eng$): macro-AUROC, macro-AUPRC, macro-precision, macro-recall, and macro-$F1$ (test, mean across folds)....7

Figure S2. Link prediction (LP AUROC) validation and test for log(DP) (top), and DP (bottom). The ranking of observed vs. mined non-edges is near ceiling for both transformations....8

Figure S3. Mean (dot) with standard deviation (whiskers) of each metric across all threshold cells. Log(DP) achieves the best downstream means; DP is nearly as strong with lower variability on most metrics; raw DP underperforms. Link-prediction AUROC is near ceiling for DP and DP and slightly lower for log(DP), indicating that downstream differences arise from DP scaling rather than pretraining quality.9

PolyRECO Visualisation Tool User Guide....13

Getting Started....13

Using the Application....14

Case Study Examples....15

Additional Experimental Details....18

NMR Spectroscopy....18

Size Exclusion Chromatography (SEC)....18

Differential Scanning Calorimetry (DSC)....18

Thermogravimetric Analysis (TGA)....18

Film Preparation....18

Tensile Testing....18

Reagents and Methods....18

Synthesis of [LZnMg($C_6F_5$)$_2$] Catalyst....19

Case Study 1 (CS-1) Polymerization Procedure....19

Case Study 2 (CS-2) Polymerization Procedure ........................................................ 19

Case Study 3 (CS-3) Polymerization Procedure ........................................................ 19

Table S1. Summary of Polymerisation Conditions and Results .................................... 21

Figure S4. $^{1}H$ NMR (400 MHz, $CDCl_3$) spectrum of case study 1 poly(cyclohexene-alt-phthalate)-b-poly(ε-caprolactone)-b-poly(cyclohexene-alt-phthalate)........................................ 21

Figure S5. $^{13}C\{^{1}H\}$ NMR (400 MHz, $CDCl_3$) spectrum of case study 1. ..................... 22

Figure S6. SEC trace (THF eluent, 1 mL $min^{-1}$) for case study 1. ................................ 22

Figure S7. DSC trace (second heating curve) for case study 1. ...................................... 23

Figure S8. TGA thermogram for case study 1 ................................................................ 23

Figure S9. $^{1}H$ NMR (400 MHz, $CDCl_3$) spectrum of case study 2 poly(cyclopentene carbonate)-b-poly(ε-decalactone)-b-poly(cyclopentene carbonate)................................................ 24

Figure S10. $^{13}C\{^{1}H\}$ NMR (400 MHz, $CDCl_3$) spectrum of case study 2.......................... 24

Figure S11. SEC trace (THF eluent, 1 mL $min^{-1}$) for case study 2................................. 25

Figure S12. DSC trace (second heating curve) for case study 2. .................................... 25

Figure S13. TGA thermogram for case study 2. ............................................................. 26

Figure S14. $^{1}H$ NMR (400 MHz, $CDCl_3$) spectrum of case study 3 poly(cyclohexene carbonate)-grad-poly(cyclopentene carbonate)-b-poly(ε-decalactone)-b-poly(cyclohexene carbonate)-grad-poly(cyclopentene carbonate). .................................................................................. 27

Figure S15. $^{13}C\{^{1}H\}$ NMR (400 MHz, $CDCl_3$) spectrum of case study 3.......................... 27

Figure S16. SEC trace (THF eluent, 1 mL $min^{-1}$) for case study 3................................. 28

Figure S17. DSC trace (second heating curve) for case study 3. .................................... 28

Figure S18. TGA thermogram for case study 3. ............................................................. 29

Table S2. Summary of Material Properties. ................................................................... 29

References ....................................................................................................................... 29

**<u>Additional Computational Details</u>**

https://github.com/SoheilaMolaei/PolyReco

**Model Description and Feature Construction**

Summary of the symbols used throughout:

| | |
|---|---|
| $\mathcal{G} = (\mathcal{V}, \mathcal{E})$ | Directed block graph; nodes are blocks, edges encode ABA order. |
| $u, v$ | Node indices (blocks). |
| $\mathbf{DP}_u$ or $\boldsymbol{d}_u$ | Degree of polymerisation (DP) of block $u$ (use one form consistently). |
| $\boldsymbol{F}_u \in \mathbb{R}^{d_F}$ | Raw block feature; $d_F$ input dimension. |
| $\bar{\boldsymbol{F}}_u$ | DP-weighted feature (e.g., $F_u\sqrt{\mathrm{DP}_u}$ or $F_u\log(\max(\mathrm{DP}_u, e))$). |
| $\boldsymbol{W}_0, \boldsymbol{W}_k$ | Learned linear projections. |
| $\boldsymbol{d}$ | Latent dimension; $K$ number of message-passing layers. |
| $\boldsymbol{h}_u^{(k)} \in \mathbb{R}^{\boldsymbol{d}}$ | Node state at layer $k$; $a_u^{(k)}$ aggregated message; $h(u) = h_u^{(K)}$. |
| $\mathbf{Nei}(\boldsymbol{u}), \mathbf{Nei}^+(\boldsymbol{u})$ | Neighbours of $u$; neighbours plus self. |
| $\mathbf{AGG}_k$ | Neighbourhood aggregation operator; $\mathrm{GAT}_k$ multi-head graph attention; LN layer norm. |
| $\mathbf{CONCAT}[\boldsymbol{x}, \boldsymbol{y}]$ **or** $\boldsymbol{x} \parallel \boldsymbol{y}$ | Concatenation; $\odot$ elementwise (Hadamard) product; $\lvert x \rvert$ elementwise absolute value. |
| $\boldsymbol{\phi}$ | Pointwise activation (e.g., ELU). |
| $\boldsymbol{s}_{\mathrm{LP}}(\boldsymbol{u}, \boldsymbol{v})$ | Bilinear LP score $h(u)^\top W_{\mathrm{LP}} h(v)$; $W_{\mathrm{LP}} \in \mathbb{R}^{d \times d}$ |
| $\mathbf{sigm}(\boldsymbol{x})$ | Logistic sigmoid $(1 + e^{-x})^{-1}$ |
| $(\boldsymbol{\ell}_\sigma, \boldsymbol{\ell}_\varepsilon)$ | Classifier logits for stress/strain; $(p_\sigma, p_\varepsilon) = \mathrm{sigm}(\ell_\sigma), \mathrm{sigm}(\ell_\varepsilon)$. |
| $\boldsymbol{\tau}_\sigma^{\mathbf{eng}}, \boldsymbol{\tau}_\varepsilon^{\mathbf{eng}}$ | Engineering thresholds; $\delta$ tolerance band. |
| $\mathbb{1}[\cdot]$ | Indicator (we use $\mathbb{1}$ consistently) |
| **TP, FP, TN, FN** | Confusion–matrix counts; precision, recall, $F_1$, AUROC, AUPRC as usual. |

**Two-Stage Learning**

**Stage 1: Link-prediction pretraining**

Given encoder states $h(u)$, a bilinear scoring head evaluates directed candidate links:

$$s_{\mathrm{LP}}(u, v) = h(u)^\top W_{\mathrm{LP}} h(v), \qquad \hat{p}_{LP}(u, v) = \mathrm{sigm}(s_{\mathrm{LP}}(u, v))$$

with $W_{\mathrm{LP}} \in \mathbb{R}^{d\times d}$. Observed edges serve as positives; non-edges are negatives, with hard negatives mined from pairs not present in $\mathcal{E}$ and ranked by similarity in the current embedding space. Training uses a margin-based ranking objective contrasting positive scores with mined negatives; model selection is by validation AUROC.

**Stage 2: Threshold-conditioned classifiers for stress and strain**

For each labelled edge $(u \rightarrow v)$, the pairwise feature

$$\mathrm{x}_{uv} = [\, h(u), h(v), h(u) \odot h(v), |h(u) - h(v)|, \hat{p}_{\mathrm{LP}}(u, v) \,]$$

is passed to a small multilayer perceptron that outputs logits $(\ell_\sigma, \ell_\varepsilon)$ indicating whether the measured stress ($\sigma_{\mathrm{break}}$) and strain ($\varepsilon_{\mathrm{break}}$) exceed engineering thresholds.

To reflect measurement variability near decision boundaries, we adopt *tolerant* labels: for target $z \in \{\sigma_{\mathrm{break}}, \varepsilon_{\mathrm{break}}\}$ and engineering threshold $\tau$,

$$y^{(\tau,\delta)} = \mathbb{1}\,[z \geq \tau - \delta],$$

where $\delta$ (task-specific) is tuned on validation to de-penalise borderline measurements. Per-task logistic losses are weighted for class imbalance.

**Graph Neural Network Processing**

The GNN processes the polymer graph through three core operations: **Initialization**, **Aggregation**, and **Update**. These operations are applied iteratively across multiple layers, allowing the model to learn progressively more informative polymer representations.

**Evaluation**

Cross-validation is performed over edges; the encoder graph and all statistics (e.g., standardization) are constructed from training folds only. We report per-task *threshold-free* metrics (AUROC/AUPRC) and *operating-point* metrics (Precision, Recall, $F_1$), each macro-averaged across the two tasks, and LP AUROC for the link-prediction head. Operating-point metrics are evaluated at task-specific probability thresholds selected on the validation split to balance precision and recall; the same thresholds are applied unchanged to test (exact rule explained below).

*Note.* Cells with $\tau_\sigma^{\text{eng}} = 0$ or $\tau_\varepsilon^{\text{eng}} = 0$ are degenerate for the corresponding task and inflate metrics; conclusions are drawn from the non-degenerate region.

**Metrics**

**Labels and scores.**

Binary labels for stress and strain are induced as above (tolerant targets around $(\tau_\sigma^{\text{eng}}, \tau_\varepsilon^{\text{eng}})$ with validation-tuned bands). For each labelled edge $(u \to v)$, the model outputs per-task probabilities $p_\sigma, p_\varepsilon \in [0,1]$ from the classifier logits via the logistic function $p_t = \text{sigm}(\ell_t)$.

**Threshold-free metrics.**

For task $t \in \{\sigma, \varepsilon\}$, AUROC is the probability that a randomly chosen positive receives a higher score than a randomly chosen negative,

$\text{AUROC}_t = \Pr(p_t^+ > p_t^-)$

AUPRC is estimated by average precision,

$$\text{AUPRC}_t \approx \sum_k Prec_t(k) \Delta Rec_t(k)$$

Macro averages are

$\text{AUROC}_{\text{macro}} = 1/2\,(\text{AUROC}_\sigma + \text{AUROC}_\varepsilon), \qquad \text{AUPRC}_{\text{macro}} = 1/2\,(\text{AUPRC}_\sigma + \text{AUPRC}_\varepsilon).$

**Operating-point metrics.**

With the usual confusion counts (TP, FP, TN, FN) on test and $\hat{y}_t = \mathbb{1}\,[\,p_t \geq \tau_t^\star\,]$,

$$\text{Precision} = \frac{\text{TP}}{\text{TP} + \text{FP}}, \qquad \text{Recall} = \frac{\text{TP}}{\text{TP} + \text{FN}}, \qquad \text{F1} = \frac{2\ \text{Precision} \cdot \text{Recall}}{\text{Precision} + \text{Recall}}.$$

Macro values average the two tasks:

$$\text{Precision}_{\text{macro}} = 1/2\,(\text{Precision}_\sigma + \text{Precision}_\varepsilon),$$

$$\text{Recall}_{\text{macro}} = 1/2\,(\text{Recall}_\sigma + \text{Recall}_\varepsilon),$$

$$\text{F1}_{\text{macro}} = 1/2\,(\text{F1}_\sigma + \text{F1}_\varepsilon).$$

**Results: Effect of chain–length weighting**

We compared two monotone transformations for incorporating chain length into block features, $\sqrt{\mathrm{DP}}$ and $\log(\mathrm{DP})$, and evaluated downstream classification of whether the measured stress and strain at break ($\sigma_{\mathrm{break}}, \varepsilon_{\mathrm{break}}$) exceed engineering targets ($\tau_\sigma^{\mathrm{eng}}, \tau_\varepsilon^{\mathrm{eng}}$). Performance is summarised across a grid of $\tau_\sigma^{\mathrm{eng}} \in \{0,10,20,30\}$ MPa and $\tau_\varepsilon^{\mathrm{eng}} \in \{0,100,\ldots,1000\}$ %. We report macro-averaged metrics across stress/strain tasks (macro-$F_1$, macro-AUPRC, macro-AUROC), and the auxiliary link-prediction AUROC (LP AUROC) (**Figure S1**).

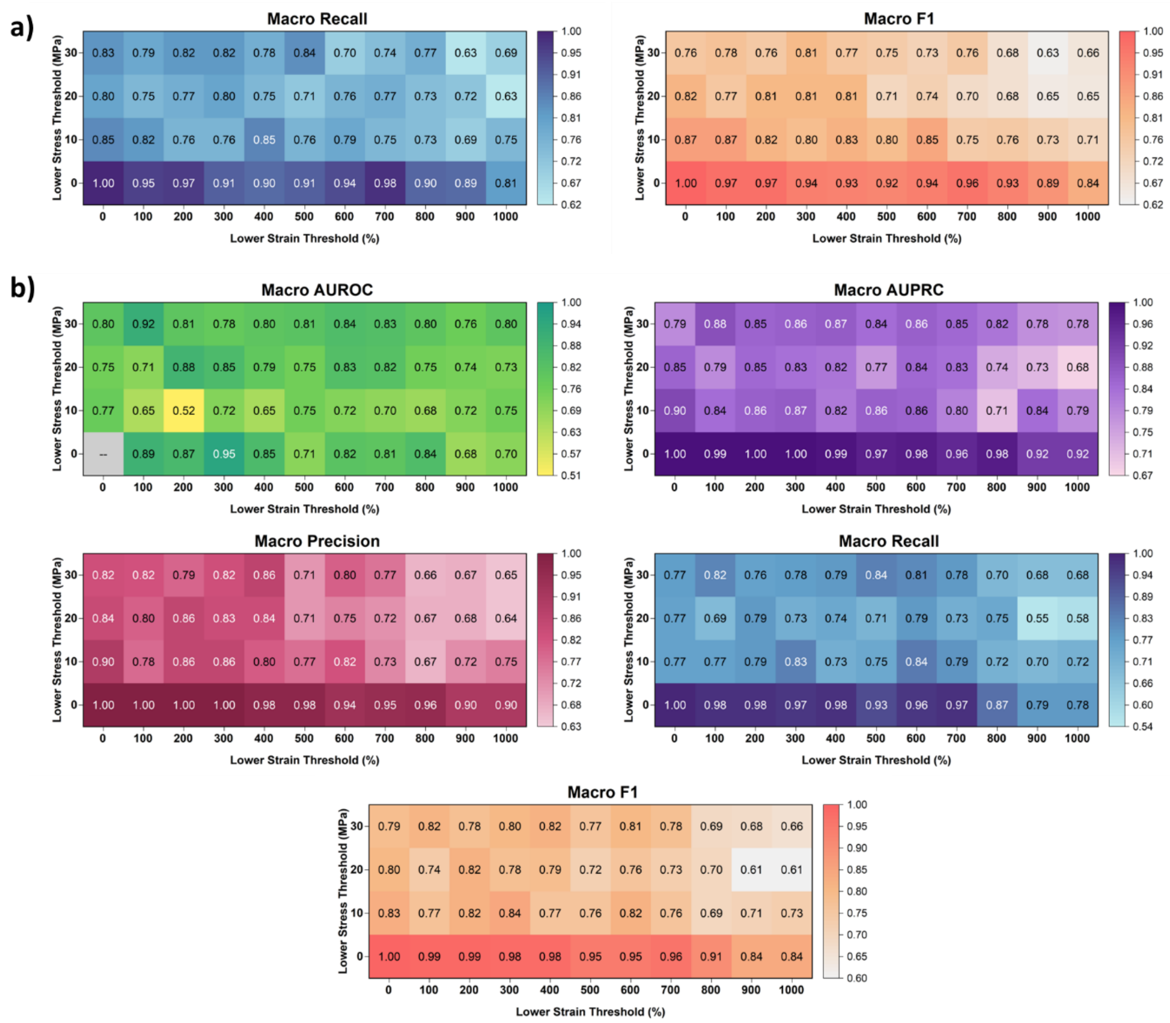


**Figure S1.** a) Log weighting ($\log(\mathbf{DP})$) across macro-recall, and macro-$\boldsymbol{F_1}$. b) Square-root weighting ($\sqrt{\boldsymbol{DP}}$) across ($\boldsymbol{\tau_\sigma^{eng}}, \boldsymbol{\tau_\varepsilon^{eng}}$): macro-AUROC, macro-AUPRC, macro-precision, macro-recall, and macro-$\boldsymbol{F_1}$ (test, mean across folds).

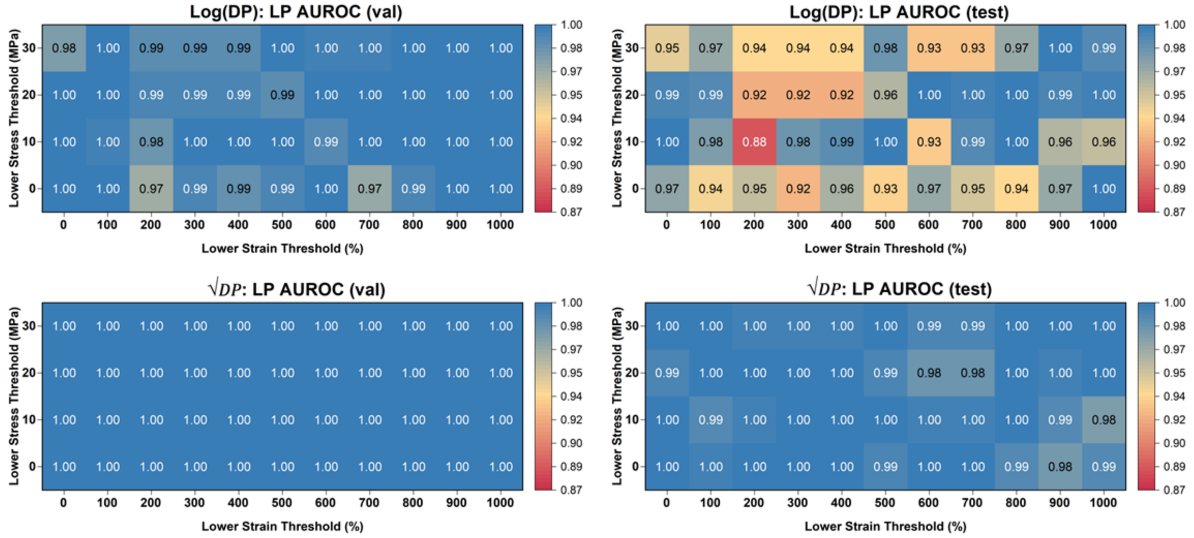


**Figure S2.** Link prediction (LP AUROC) validation and test for $\mathbf{log(DP)}$ (top), and $\sqrt{\mathbf{DP}}$ (bottom). The ranking of observed vs. mined non-edges is near ceiling for both transformations.

### Implications for polymer design

The two weightings emphasise different practical objectives:

- *Prioritising synthesizability and high hit precision* (e.g., down-selecting a small set of formulations for tensile testing at moderate targets): use log(DP). It achieves consistently higher macro-precision and strong macro-F1 where many engineering polymers operate ($\tau_\sigma^{eng}$ = 10–20 MPa, $\tau_\varepsilon^{eng}$ = 400–700 %).
- *Aggressive exploration for high-strength elastomers* (e.g., seeking rare formulations achieving $\tau_\sigma^{eng}$ = 30 MPa while maintaining ductility): use $\sqrt{\text{DP}}$. It delivers higher recall at strict $\tau_\sigma^{eng}$ with competitive AUPRC, reducing false negatives (missed candidates) when high-strength positives are scarce.

In both modes, the LP head reliably filters implausible block orders, so laboratory effort is focused on chemically compatible A–B combinations; the choice of DP transform then tunes the risk profile between precision-oriented down-selection and recall-oriented exploration.

### Across-grid meta-summary

In **Figure S3**, the averages of each metric over the full threshold grid are presented. On downstream metrics, log(DP) yields the best means (macro-F1 = 0.80 ± 0.10, AUPRC 0.87 ± 0.08, AUROC 0.80±0.06); $\sqrt{\text{DP}}$ is a close second (F1 = 0.80 ± 0.08, 0.86 ±0.07, 0.77±0.08) with lower variability on

F1, precision, recall, and AUPRC. Raw DP underperforms on mean and stability. LP AUROC is near ceiling for $\sqrt{\text{DP}}$ and DP (val 1.000, test 0.996 ± 0.006); log(DP) is slightly lower (val 0.995 ± 0.008, test 0.963 ± 0.030) yet still delivers the strongest downstream means, indicating that the downstream gains are driven by DP scaling rather than LP pretraining quality. Both transforms are effective: log(DP) excels at high-precision ranking near commonly used stress/strain targets, whereas $\sqrt{\text{DP}}$ favours sensitivity at stringent strength thresholds. These complementary behaviours provide a principled knob for aligning the model with experimental priorities.

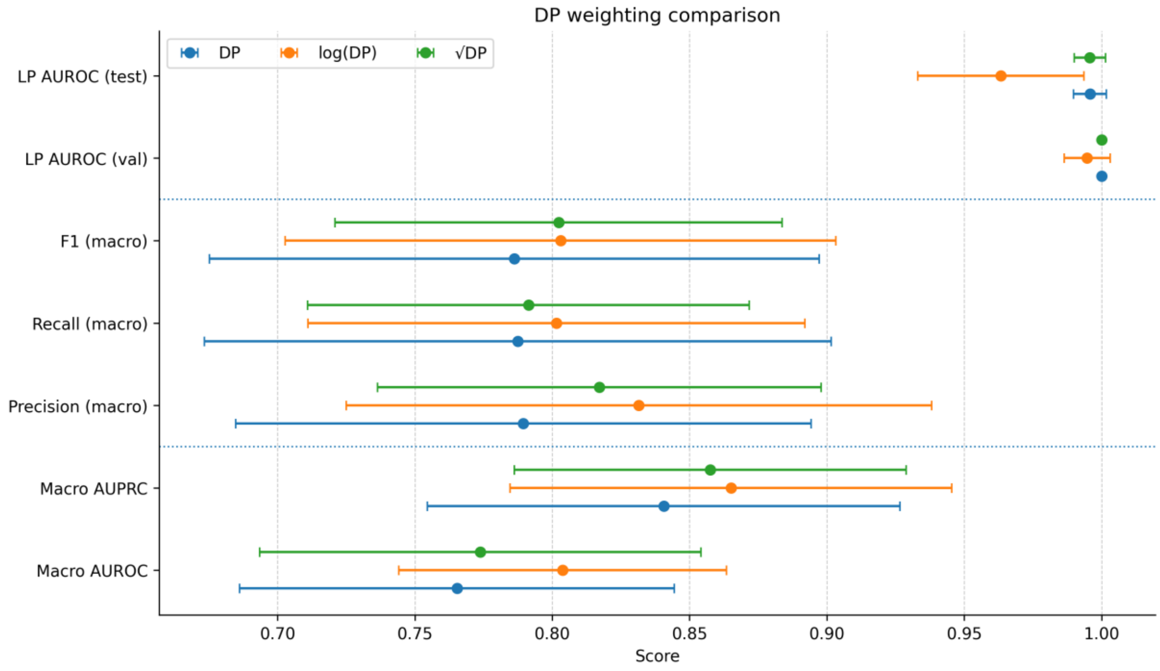


**Figure S3.** Mean (dot) with standard deviation (whiskers) of each metric across all threshold cells. **Log(DP)** achieves the best downstream means; $\sqrt{\textbf{DP}}$ is nearly as strong with lower variability on most metrics; raw DP underperforms. Link-prediction AUROC is near ceiling for $\sqrt{\textbf{DP}}$ and DP and slightly lower for **log(DP)**, indicating that downstream differences arise from DP scaling rather than pretraining quality.

### Supplementary Note: Why concave chain–length weighting is preferable to linear DP

*Setting and notation.* Each block $u$ has a chemistry feature $F_u \in \mathbb{R}^{d_F}$ and a DP $d_u > 0$. The initial states are:

$$h_u^{(0)} = W_0 \left(F_u\, f(d_u)\right)$$

where $W_0 \in \mathbb{R}^{d \times d_F}$ is linear and $f : (0, \infty) \to \mathbb{R}_+$ is increasing and concave. We study (i) the power family $f_\alpha(d) = d^\alpha$ with $\alpha \in (0,1]$ ($\alpha = \frac{1}{2}$ gives $\sqrt{d}$; $\alpha = 1$ is linear DP), and (ii) $f_{\log}(d) = \log(\max\{d, e\}$ (the clamp at $e$ avoids undefined values and bounds the derivative near 0). A K–layer message-passing encoder $E$ (graph attention) maps $h^{(0)} \mapsto h^{(K)} =: h(\cdot)$. Let $L_{\text{enc}}$ denote a global

Lipschitz constant of $E$ on bounded inputs (well-known for residual MPNNs with bounded weights/softmax).

*Standing assumptions.*

A1. Bounded chemistry. $\| F_u \| \leq B$ for all $u$ (finite WL/RDKit/embedding range).

A2. Multiplicative dispersity noise. The observed DP is $d_{\text{obs}} = d\ \xi$ with $\xi > 0$ and $log\ \xi$ having mean 0 and variance $\sigma^2(d)$; for small dispersity, $Var(log\xi) = \sigma^2 \ll 1$.

A3. Encoder Lipschitzness. For any two initial tensors $z, z'$ on the same graph, $\| E(z) - E(z') \| \leq L_{\text{enc}} \| z - z' \|$.

**A. Deterministic properties of concave *f***

*Lemma 1 (Scale equivariance and identity preservation).* For fixed chemistry $F_u$, $h_u^{(0)}(d') = \frac{f(d')}{f(d)} h_u^{(0)}(d)$. Thus, DP acts as a scalar gain on an invariant chemical identity; concavity implies diminishing returns (the gain ratio grows sublinearly with $d$).

Immediate from linearity: $h_u^{(0)}(d') = W_0\big(F_u f(d')\big) = \frac{f(d')}{f(d)} W_0(F_u f(d))$

<u>Theorem 1 (Dynamic-range contraction).</u> Let $0 < d_{\min} \leq d \leq d_{\max}$ and $f$ be increasing and concave with $f(0) \geq 0$. Then

$$\frac{f(d_{\max})}{f(d_{\min})} \leq \frac{d_{\max}}{d_{\min}}$$

Consequently, for $f_\alpha(d) = d^\alpha$,

$\frac{f_\alpha(d_{\max})}{f_\alpha(d_{\min})} \leq \left(\frac{d_{\max}}{d_{\min}}\right)^\alpha \leq \frac{d_{\max}}{d_{\min}} \quad (\alpha < 1),$

and for $f_{\log}$ with the stated clamp, $\frac{f_{\log(d_{\max})}}{f_{\log(d_{\min})}} \leq \log d_{\max}$.

For concave $f$, the map $d \mapsto f(d)/d$ is nonincreasing on $(0,\infty)$ (secant slopes decrease). Hence, $\frac{f(d_{\max})}{d_{\max}} \leq \frac{f(d_{\min})}{d_{\min}}$, which rearranges to the claim. The power/log cases follow directly.

*Interpretation.* Relative to linear DP, any concave transform reduces the DP-induced spread of feature norms across the dataset. This improves numerical conditioning, gradient stability, and the reliability of fold-wise standardisation (computed on train only).

**B. Noise stabilisation under dispersity**

We now quantify how multiplicative DP noise propagates to the encoder input and output.

Theorem 2 (First-order noise sensitivity). Let $x(d) := W_0(Ff(d))$ with $\| F \| \leq B$ and $d_{\text{obs}} = d\,\xi$ as in (A2). For small $\sigma^2 = \text{Var}(\log \xi)$, the delta method gives

$$\mathbb{E} \| x(d_{\text{obs}}) - x(d) \|^2 = \| W_0 \|^2 \| F \|^2 (f'(d)d)^2\sigma^2 + o(\sigma^2).$$

Therefore, the input-level noise amplification scales with $(f'(d)\,d)^2$. For $f_\alpha(d) = d^\alpha, f'(d)\,d = \alpha d^\alpha$ so the amplification is $\propto d^{2\alpha}$; for $f_{\log}$, $f'(d)\,d \equiv 1$ (away from the clamp), i.e. essentially constant in $d$.

Let $\varepsilon = \log\xi$ with $\mathbb{E}[\varepsilon] = 0$, $\text{Var}(\varepsilon) = \sigma^2$. Define $\varphi(\varepsilon) = f(de^\varepsilon)$; then $\varphi'(0) = \frac{\partial}{\partial\varepsilon} f(de^\varepsilon)|_{\varepsilon=0} = f'(d)\,d$. By the delta method, $f(de^\varepsilon) - f(d) = \varphi'(0)\,\varepsilon + o(\varepsilon)$ in $L_2$, so $\mathbb{E}|f(d\xi) - f(d)|^2 = (f'(d)d)^2\sigma^2 + o(\sigma^2)$. Multiply by $\| W_0F \|^2$ and use $\| W_0F \| \leq \| W_0 \| \| F \|$.

[End-to-end stability] With (A3),

$$\mathbb{E} \| h(d_{\text{obs}}) - h(d) \|^2 \leq L_{\text{enc}}^2 \| W_0 \|^2 B^2(f'(d)d)^2 \sigma^2 + o(\sigma^2)$$

Hence, $\sqrt{\text{DP}}$ $(\alpha = 1/2)$ and $\log(\text{DP})$ propagate significantly less DP noise than linear DP $(\alpha = 1)$, especially at large $d$. Apply Theorem 2 at input and (A3) for the encoder.

**C. Capacity and generalisation**

We now relate concavity to effective capacity and sample complexity.

Theorem 3 (Rademacher complexity bound). Let $\mathcal{H}$ be any class of linear predictors on the encoder outputs $h(u)$ with parameter norm bounded by $C$ (e.g., the first affine layer of the stage-2 MLP), and let $d \in [d_{\min}, d_{\max}]$. Then, for a sample of size $n$,

$$\mathfrak{R}_n(\mathcal{H} \circ E) \leq \frac{C\,L_{\text{enc}} \| W_0 \|}{\sqrt{n}} \left(\max_u \| F_u \|\right) f(d_{\max})$$

For $f_\alpha$, this is $O(d_{\max}^{\alpha}/\sqrt{n})$; for $f_{\log}$, it is $O(d_{\max}/\sqrt{n})$. Thus, concave transforms strictly tighten the bound versus linear DP ($(\alpha = 1)$.

Let $x_i = W_0\left(F_{u_i} f\left(d_{u_i}\right)\right)$ and $z_i = E(x_i)$. For linear $\| w \| \leq C$, the empirical Rademacher complexity satisfies $\mathfrak{R}_n \leq \frac{C}{n}\ \mathbb{E} \parallel \sum_{i=1}^{n} \epsilon_i z_i \parallel \leq \frac{C}{n}\ \mathbb{E} \sum_i \parallel z_i \parallel \leq \frac{C}{\sqrt{n}} \max_i \parallel z_i \parallel$. By (A3), $\| z_i \| \leq L_{\text{enc}} \| x_i \| \leq L_{\text{enc}} \| W_0 \| \| F_{u_i} \| f(d_{u_i}) \leq L_{\text{enc}} \| W_0 \| (\max_u \| F_u \|) f(d_{\max})$.

Theorem 4 (Covariance conditioning w.r.t. DP spread). Consider any finite dataset $\{(F_i, d_i)\}_{i=1}^{n}$ with $d_i \in [d_{\min}, d_{\max}]$ and form the matrix $X_f = [\, W_0 F_i f(d_i)\, ]_{i=1}^{n}$. Let $\kappa(\cdot)$ denote the spectral condition number. Then

$$\kappa(X_{f_\alpha}) \leq \kappa(X_{\text{chem}})\ (\frac{d_{\max}}{d_{\min}})^{\alpha}, \quad \kappa(X_{\text{lin}}) \leq \kappa(X_{\text{chem}})\ \left(\frac{d_{\max}}{d_{\min}}\right),$$

where $X_{\text{chem}} = [\, W_0 F_i\, ]_{i=1}^{n}$. Thus, any $\alpha < 1$ reduces the worst-case DP-induced ill-conditioning by a factor $(d_{\max}/d_{\min})^{1-\alpha}$.

Right-multiplication by $\text{Diag}(f(d_1), \ldots, f(d_n))$ scales each column. For any matrix $A$ and diagonal $D, \kappa(AD) \leq \kappa(A)\ \frac{\max_j |D_{jj}|}{\min_j |D_{jj}|}$. Apply with $A = X_{\text{chem}}$ and $D = \text{Diag}(f(d_i))$ and use Theorem 1.

*Remarks on standardisation.* Per-fold $z$-scoring is an affine reparameterisation and does not affect the ordering implied by Theorems 2–4. Concave $f$ reduces heteroskedasticity before normalisation, yielding more stable empirical moments and better-behaved attention weights during training.

**D. Consequences for $\sqrt{\text{DP}}$ and $\log(\text{DP})$**

Combining Theorems 2-4 yields:

- *Noise robustness.* Input and end-to-end perturbations due to dispersity scale as $(f'(d)d)^2$; hence DP (linear) $\propto d^2$, $\sqrt{\text{DP}} \propto d$, $\log(\text{DP}) \approx$ const.
- *Capacity/conditioning.* Effective capacity and worst-case condition number grow with $f(d_{\max})$ and $f(d_{\max})/f(d_{\min})$, respectively; concave $f$ strictly improves both relative to linear DP, with log compressing the most and $\sqrt{\cdot}$ retaining more DP signal.

These mathematical advantages match the empirical pattern in the heatmaps: $\log(\mathrm{DP})$ favours precision (strong compression, tight capacity/conditioning), while $\sqrt{\mathrm{DP}}$ preserves more chain-length signal, boosting recall at stricter stress targets, all without sacrificing robustness to DP noise.

## PolyRECO Visualisation Tool User Guide

### PolyReco Visualization Tool (v1.0.0):

https://github.com/GeorgeLGregory/PolyRECO-Visualization-Tool

#### Overview

The PolyRECO Visualization Tool is a MATLAB application designed to help explore and visualize potential polymer combinations that meet specific mechanical thresholds. While PolyRECO performs the machine learning analysis, this visualization tool allows the user to interact with the results in an intuitive way.

#### What the Tool Does

The visualization tool converts SMILES strings into familiar chemical names and allows the user to explore different polymer combinations based on various parameters such as:

- Degree of polymerization (DP)
- Glass transition temperature ($T_g$)
- ABA Block Weight percentages
- Copolymer compositions

#### Naming Convention

Polymer names follow this format:

- ROCOP polyesters: Epoxide_Anhydride
- ROCOP polycarbonates: Epoxide_CO2
- Copolymer blocks are indicated with a comma (,). e.g. CHO_CO2,CPO_CO2

## Getting Started

#### Requirements

1. PolyRecoVisualisationTool.App (from the provided zip file)
2. MATLAB Runtime environment (no full MATLAB license needed)
3. Case study files (Case1-3.xls) and Mapping.xlsx file

**Installation and Setup**

1. Download and install the PolyRecoVisualisationTool.App from the zip file
2. Ensure you have the MATLAB installer or runtime environment
3. Keep the case study files and mapping file accessible

**<u>Using the Application</u>**

**Data Loading and Processing**

1. **Load Case Study:** Click the yellow "LoadData" button and select a case file (e.g., case1.xls). A confirmation message will appear when successful.
2. **Load Mapping File:** Click the yellow "LoadMapping" button and select the Mapping.xls file. A confirmation message will appear when successful.
3. **Convert SMILES to Names:** Click the pink "SMILEStoNAME" button to update the table with chemical names corresponding to the SMILES strings.
4. **Calculate Weight Percentages:** Click the purple "Calculate wt%" button. The green indicator light will turn red during calculation (which takes a few minutes). Once complete, the light will return to green and a confirmation message will appear. The "A_Block_WtPercent" column will be added to the table.
5. **Save Your Work:** Use the "SaveTable" button to save the processed data for future use.

**Visualization and Analysis**

1. **Select Starting Block:** Use the toggle switch to choose whether to start with A or B Block.
2. **Select Polymer:** Once A or B Block is selected, the Polymer Name dropdown (blue) will show all polymer options that meet the mechanical thresholds. You can refine this list using the $T_g$ Range Slider.
3. **Adjust DP Range:** Use the DP slider to select the degree of polymerization values. Note that the slider allows a range of up to 50 units to account for polymer dispersity.
4. **Explore Partner Options:** The bar chart will display all partner block options for your chosen polymer and DP range. The X-axis shows partner block names. For copolymers, a ratio (0.x) indicates the proportion of the first named block. When selecting a copolymer block, an additional dropdown will appear allowing you to specify the copolymer composition.
5. **Customize the Visualization:** Bar labels can show: partner DP, weight percentage (wt%), or glass transition temperature ($T_g$). Y-axis options include: Count (number of occurrences), DP, or wt%. Values shown for DP and wt% are averages for the partner blocks within the selected DP range. Note: wt% calculations must be performed (step 4) before they can be displayed

<u>**Case Study Examples**</u>

## Case Study 1

1. Load and map case1.xls
2. Select B Block
3. Choose εCL (epsilon-caprolactone) from the dropdown. Note: The $T_g$ Range automatically updates to show poly(εCL) typically has $T_g$ values of -64 to -59°C.
4. Set the DP range below 500
5. The bar chart will show various partner A blocks including poly(CHO-*alt*-PA) with an average DP of 39

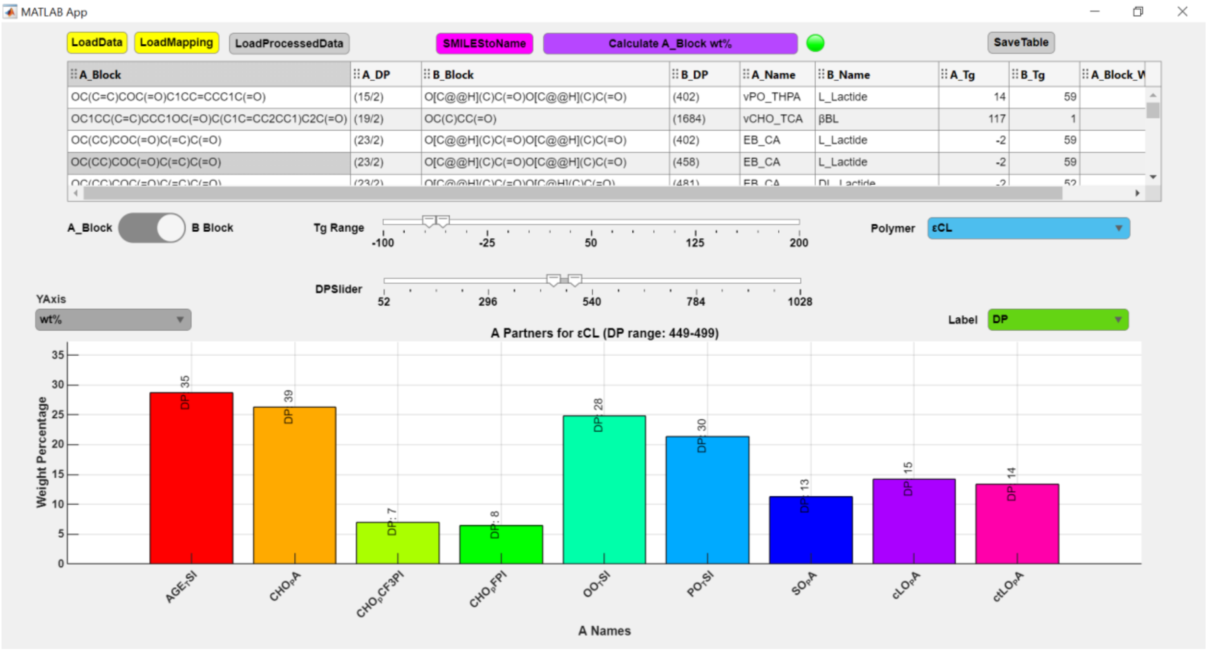


## Case Study 2

1. Load and map case2.xls
2. Select B Block
3. Adjust the $T_g$ range to below -25°C to filter for low-$T_g$ B blocks
4. Select poly(εDL) from the options
5. The bar chart will show all A block partners with low A block wt% (<22 wt%) for hard (high $T_g$) polymers, consistent with materials expected to give mechanical properties in this range.

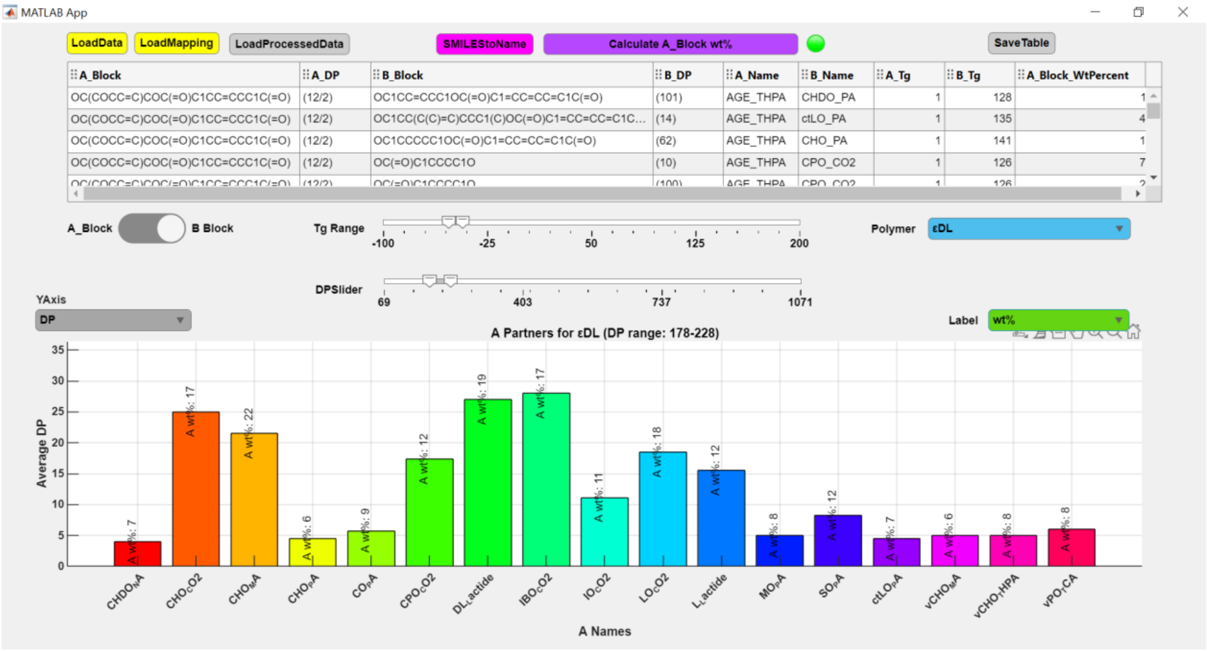


## Case Study 3

1. Load and map case3.xls
2. Select A Block
3. Filter for high $T_g$ (>100°C) as is typical for polystyrene A blocks in SBS
4. Select CHO_CO2, CPO_CO2
5. Choose the ratio of CHO-*alt*-$CO_2$ to CPO-*alt*-$CO_2$ before selecting the total DP (a ratio of 0.6 is PCHC:PCPC of 48:30).
6. The bar chart will update to show partner blocks for the copolymer composition and DP range selected.

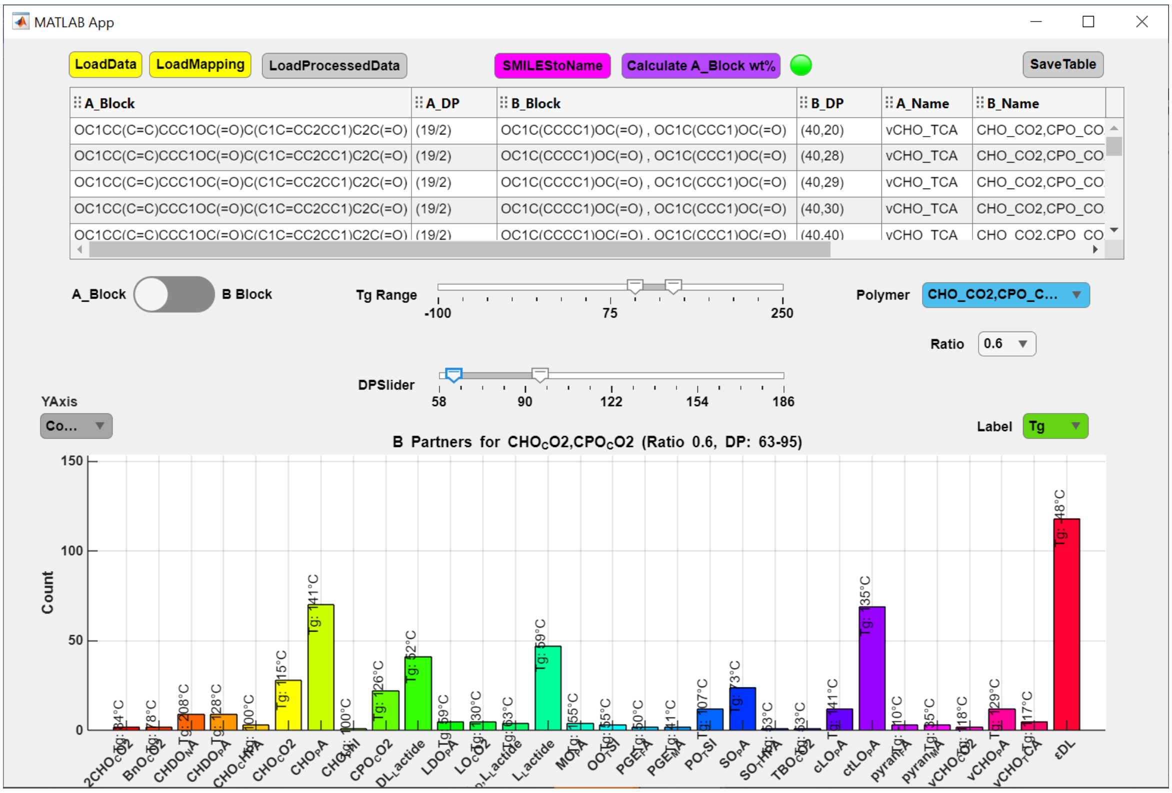
MATLAB App
LoadData
LoadMapping
LoadProcessedData
SMILEStoName
Calculate A_Block wt%
SaveTable
A_Block
A_DP
B_Block
B_DP
A_Name
B_Name
OC1CC(C=C)CCC1OC(=O)C(C1C=CC2CC1)C2C(=O)
(19/2)
OC1C(CCCC1)OC(=O) , OC1C(CCC1)OC(=O)
(40,20)
(40,28)
(40,29)
(40,30)
vCHO_TCA
CHO_CO2,CPO_CO
A_Block
B Block
Tg Range
-100
75
250
Polymer
CHO_CO2,CPO_C...
Ratio
0.6
DPSlider
58
90
122
154
186
YAxis
Co...
Label
Tg
B Partners for CHO$_C$O2,CPO$_C$O2 (Ratio 0.6, DP: 63-95)
Count
150
100
50
0
εDL

## Additional Experimental Details

### NMR Spectroscopy

$^1$H NMR spectra were obtained using a Bruker AVIII HD 400 NMR spectrometer. $^{13}$C{$^1$H} NMR spectra were obtained using a Bruker NEO 600 NMR spectrometer.

### Size Exclusion Chromatography (SEC)

Polymers (2-5 mg) were dissolved in THF. Samples were passed through 0.2 μm PTFE filters prior to analysis. Analysis was carried out on an Agilent LC1260 Infinity II system instrument, fitted with a a PLgel 5 μm (50 x 7.5 mm) guard column, two PLgel 5 μm MIXED-C (300 x 7.5 mm) analytical columns and equipped with a multi-detector suite (MDS) comprising a dual-angle light scattering detector (LS, 15 & 90 degrees), refractive index detector (RI), and viscometer (VS). THF (FisherScientific, GPC grade stabilized with 0.025% BHT) was used as the eluent, with a flow rate of 1 mL min$^{-1}$, at 35 °C. The system was calibrated using a set of narrow polystyrene standards (Agilent EasiVial PS-H 2 mL) for standard GPC calibration and a narrow polystyrene standard ($M_p$ = 5 29,510 g/mol, $M_w$, LS = 29,810 g/mol, dn/dc = 0.185, $M_w/M_n$ = 1.02, [η] = 0.1777 dL/g) for system calibration and triple detection.

### Differential Scanning Calorimetry (DSC)

Recorded for purified polymer samples of triblock copolymers were measured using a DSC25 (TA Instruments). A sealed, empty crucible was used as a reference, and the DSC was calibrated using sapphire and indium. Samples were heated from -80 °C to 150 °C, at a rate of 10 °C min$^{-1}$, under $N_2$ flow (80 mL min$^{-1}$), followed by a 5-minute isotherm, at 150 °C, to erase thermal history. Samples were subsequently cooled to -80 °C, at a rate of 10 °C min$^{-1}$, and kept at -80 °C for a further 5 minutes, followed by a heating-cooling procedure from -80 °C to 150 °C, at a rate of 10 °C min$^{-1}$. Each sample was analysed over two heating-cooling cycles. Glass transition temperatures ($T_g$) are reported as the midpoint of the transition taken from the second heating cycle.

### Thermogravimetric Analysis (TGA)

Measured using a TGA5500 system (TA Instruments). Samples were heated from 30 °C to 600 °C, at a rate of 5 °C min$^{-1}$, under $N_2$ flow (100 cm$^3$ min$^{-1}$).

### Film Preparation

For mechanical testing, transparent films were prepared by solvent casting into Teflon moulds from tetrahydrofuran solutions. The solvent allowed to dry at room temperature, prior to being dried in a vacuum oven, at 60 °C, for at least 48 hours or until no solvent was observed by NMR or TGA analysis.

### Tensile Testing

Tests were carried out using a universal testing machine (Instron 6800). Dumbbell-shaped specimens were cut using a Zwick ZCP020 cutting press, equipped with a cutting device for ISO 527-2 type 5B (length= 35 mm, gauge length = 10 mm, width = 2 mm). Uniaxial extension experiments (10 mm min$^{-1}$ cross-head speed) were run according to ISO 527. 5 specimens were tested for each material.

## Reagents and Methods

The macrocyclic ligand, $H_2L$, was synthesized following a previously reported procedure.[1] Magnesium bis(1,1,1,3,3,3-hexamethyldisilazan-2-ide) (97%) and bis(pentafluorophenyl)zinc (97%) were purchased from Sigma-Aldrich. Bis(pentafluorophenyl)zinc was used as received and magnesium bis(1,1,1,3,3,3-hexamethyldisilazan-2-ide) was recrystallized from hexane. Solvents used for synthesis and polymerization were collected from a solvent purification system (SPS), degassed with three freeze-pump-thaw cycles, and stored over 4 Å molecular sieves, under an inert atmosphere. ε-Decalactone (ε-DL) (Sigma-Aldrich, 98% purity) was dried over $CaH_2$ followed by fractional distillation at 120 °C under reduced pressure and stored

under a nitrogen atmosphere. ε-Caprolactone (ε-CL) (Sigma-Aldrich, 98% purity) was dried over $CaH_2$ followed by fractional distillation at 120 °C under reduced pressure and stored under a nitrogen atmosphere. Cyclohexene 1,2-epoxide (98 %) (CHO) was purchased from Alfa Aesar, dried by stirring over $CaH_2$, followed by fractional distillation at 60 °C. Cyclopentene 1,2-epoxide (98 %) (CPO) was purchased from Sigma-Aldrich, dried by stirring over $CaH_2$, followed by fractional distillation at 60 °C. Phthalic anhydride (PA) (Sigma Aldrich, anhydrous, 98% purity) was stirred in dry toluene (purified by SPS) for 16 h. After cannula filtration and removal of toluene *in vacuo*, the white powder was crystalised from anhydrous chloroform and sublimed three times, under vacuum, at 80 °C, then stored in a glovebox. 1,4-Benzenedimethanol (BDM) was crystallized from toluene and stored under inert atmosphere.

**Synthesis of [LZnMg($C_6F_5$)$_2$] Catalyst**

The catalyst was synthesized following a previously reported procedure.[1] Under inert conditions, the macrocyclic ligand ($H_2L$) (0.50 g, 0.90 mmol) and recrystallised Mg{N[Si($CH_3$)$_3$]$_2$}$_2$ (0.31 g, 0.90 mmol) were dissolved in THF (10 mL), at 25 °C, for 1 hour. Zn($C_6F_5$)$_2$ (0.36 g, 0.90 mmol) was dissolved in THF (5 mL) and added dropwise to the reaction mixture to afford an off-white solution which was stirred overnight at 25 °C. Solvent was removed *in vacuo* to yield an off-white solid. The product was isolated by washing with cold (-30 °C) THF (5 mL) and pentane (2 x 5 mL) followed by centrifugation. The off-white powder was dried *in vacuo* overnight at 25 °C (0.62 g, 70% yield). $^1$H NMR (400 MHz, $CDCl_3$) δ 6.81 (s, 2H), 6.76 (s, 2H), 4.42 – 4.25 (m, 4H), 3.40 (d, J = 13.7 Hz, 2H), 3.26 (d, J = 13.3 Hz, 2H), 3.14 – 2.94 (m, 4H), 2.68 (s, 6H), 2.11 – 2.00 (m, 2H), 1.27 (s, 3H), 1.20 (s, 18H), 1.17 (s, 3H), 1.06 – 1.01 (m, 6H).

**Case Study 1 (CS-1) Polymerization Procedure**

In a glovebox, ε-CL (4.54 mL, 41.0 mmol, 1000 equiv.) was added to a solution of CHO (0.96 mL, 9.52 mmol, 463 equiv.), 1,4-BDM (5.0 mg, 0.0410 mmol, 2 equiv.), and [LZnMg($C_6F_5$)$_2$] (20 mg, 0.0205 mmol, 1 equiv.) in toluene (40 mL). The reaction vessel was sealed and heated to 80 °C. The progress of the reaction was monitored by aliquot removal and analysis by $^1$H NMR spectroscopy. After 1 hour, >95% conversion of lactone was achieved. PA (0.94 g, 15.8 mmol, 309 equiv.) was added to the reaction mixture, then heated to 110 °C and stirred for 72 hours. PA conversion was determined by NMR spectroscopy of an aliquot (>99%). The reaction mixture was quenched by exposure to air. The polymer was purified by precipitation in methanol (3 x 400 mL) and dried under reduced pressure to remove all solvents. $^1$H NMR (400 MHz, $CDCl_3$) δ 7.58 (m, 2H, d), 7.40 (m, 2H, e), 5.14 (br s, 2H, a), 4.06 (m, 2H, f), 2.30 (m, 2, j), 1.77-1.34 (br m, 10H, b+c+g+h+i). Integrals given are for a single repeat unit of each block. SEC (THF eluent, 1 mL min$^{-1}$) $M_n$ = 76.4 kg mol$^{-1}$, $Đ$ = 1.44.

**Case Study 2 (CS-2) Polymerization Procedure**

In a glovebox, ε-DL (2.94 mL, 16.8 mmol, 1645 equiv.) was added to a solution of CPO (0.74 mL, 9.61 mmol, 96 equiv.), 1,4-BDM (5.7 mg, 0.0412 mmol, 4 equiv.), and [LZnMg($C_6F_5$)$_2$] (10 mg, 0.0103 mmol, 1 equiv.) in toluene (10 mL). The reaction vessel was sealed and heated to 80 °C. The progress of the reaction was monitored by aliquots which were analysed by $^1$H NMR spectroscopy. After 1 hour, >80% conversion of lactone was achieved. The reaction was placed under 20 bar of $CO_2$, in a high-pressure reactor, heated to 80 °C and left to stir overnight. The reaction mixture was quenched by exposure to air. The polymer was purified by precipitation in methanol (3 x 400 mL) and dried under reduced pressure to remove all solvents. $^1$H NMR (400 MHz, $CDCl_3$) δ 5.01 (br s, 2H, a), 4.85 (m, 1H, d), 2.27 (m, 2H, h), 2.14 (br s, 2H, b+c), 1.77-1.28 (br m, 12H, e+f+g+i+j+k), 0.88 (t, 3H, l). Integrals given are for a single repeat unit of each block. SEC (THF eluent, 1 mL min$^{-1}$) $M_n$ = 51.3 kg mol$^{-1}$, $Đ$ = 1.20.

**Case Study 3 (CS-3) Polymerization Procedure**

In a glovebox, ε-DL (2.94 mL, 16.8 mmol, 1645 equiv.) was added to a solution of CPO (0.74 mL, 9.61 mmol, 96 equiv.), CHO (0.97 mL, 9.61 mmol, 96 equiv.), 1,4-BDM (5.7 mg, 0.0412 mmol, 4 equiv.), and [LZnMg($C_6F_5$)$_2$] (10 mg, 0.0103 mmol, 1 equiv.) in toluene (10 mL). The reaction vessel was sealed and

heated to 80 °C. The progress of the reaction was monitored by aliquots which were analysed by $^1$H NMR spectroscopy. After 1 hour, >80% conversion of lactone was achieved. The reaction was placed under 20 bar of $CO_2$, in a high-pressure reactor heated to 80 °C, and left to stir overnight. The reaction mixture was quenched by exposure to air. The polymer was purified by precipitation in methanol (3 x 400 mL) and dried under reduced pressure to remove all solvents. $^1$H NMR (400 MHz, $CDCl_3$) δ 5.00 (br s, 2H, d), 4.85 (m, 1H, d), 4.64 (br s, 2H, a), 2.27 (m, 2H, k), 2.14 (br s, 4H, b+c+e+f), 1.78-1.29 (br m, 12H, h+i+j+l+M+n), 0.88 (t, 3H, o). Integrals given are for a single repeat unit of each block. SEC (THF eluent, 1 mL min$^{-1}$) $M_n$ = 65.5 kg mol$^{-1}$, $Đ$ = 1.18.

**Table S1.** Summary of Polymerisation Conditions and Results

| # | Lactone ROP | | Epoxide/heterocumulene ROCOP | | $M_{n\ SEC}$[b] [Đ][c] (kg mol$^{-1}$) | Outer block (wt%) |
|---|---|---|---|---|---|---|
| | Time (h) | Conv.[a] (%) | Time (h) | Conv.[a] (%) | | |
| **CS-1** | 0.5 | 99 | 72 | 99 | 76.4 [1.5] | 27 |
| **CS-2** | 2.0 | 83 | 72 | 67 | 51.3 [1.2] | 18 |
| **CS-3** | 2.0 | 81 | 72 | 91 | 65.5 [1.18] | 40 |

*[a] Determined from $^1H$ NMR spectrum of reaction aliquot. [b] Determined from SEC analysis (THF, 1mL min$^{-1}$), RI and UV detector, calibrated using poly(styrene) standards. [c] $M_w/M_n$. [d] Determined from $^1H$ NMR sample of purified polymer sample.*

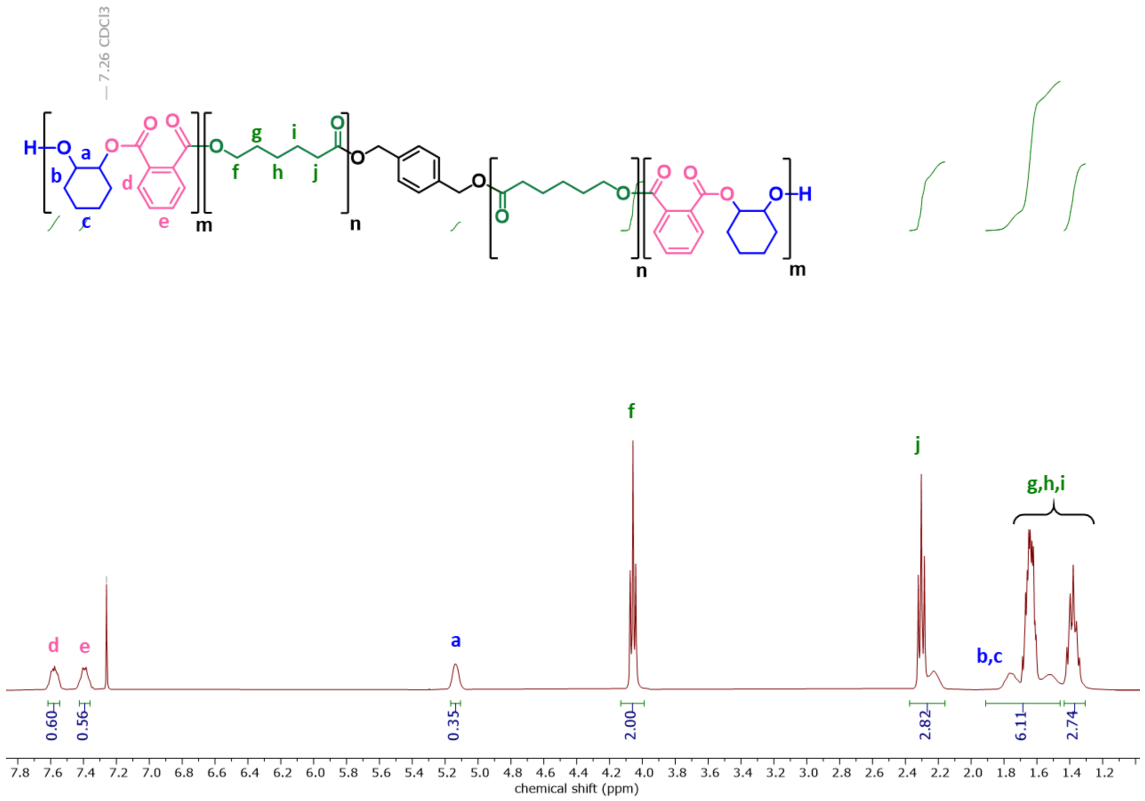


**Figure S4.** $^1H$ NMR (400 MHz, $CDCl_3$) spectrum of case study 1 poly(cyclohexene-alt-phthalate)-b-poly(ε-caprolactone)-b-poly(cyclohexene-alt-phthalate).

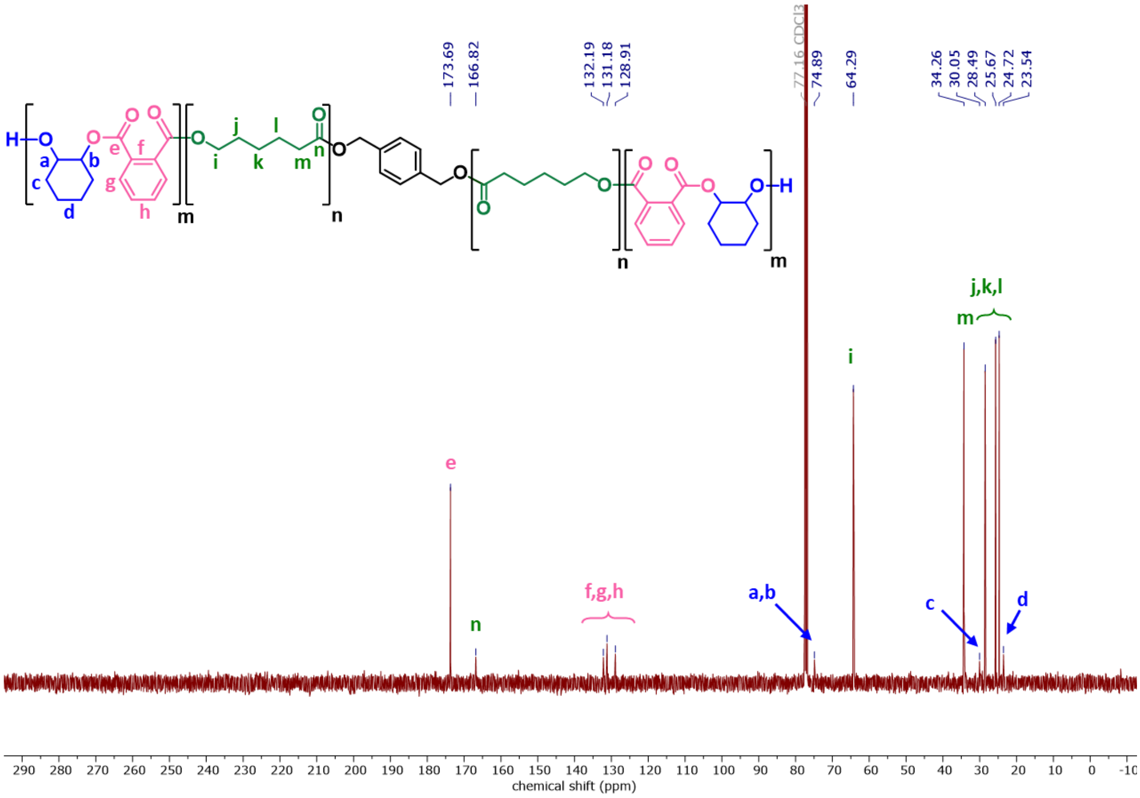


**Figure S5.** $^{13}C\{^{1}H\}$ NMR (400 MHz, $CDCl_3$) spectrum of case study 1.

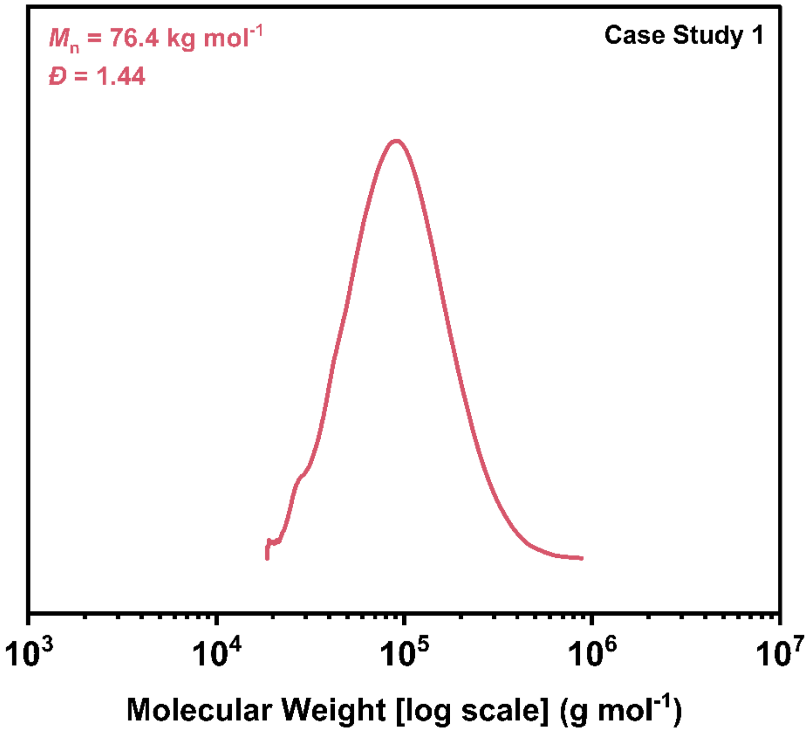


**Figure S6.** SEC trace (THF eluent, 1 mL min$^{-1}$) for case study 1.

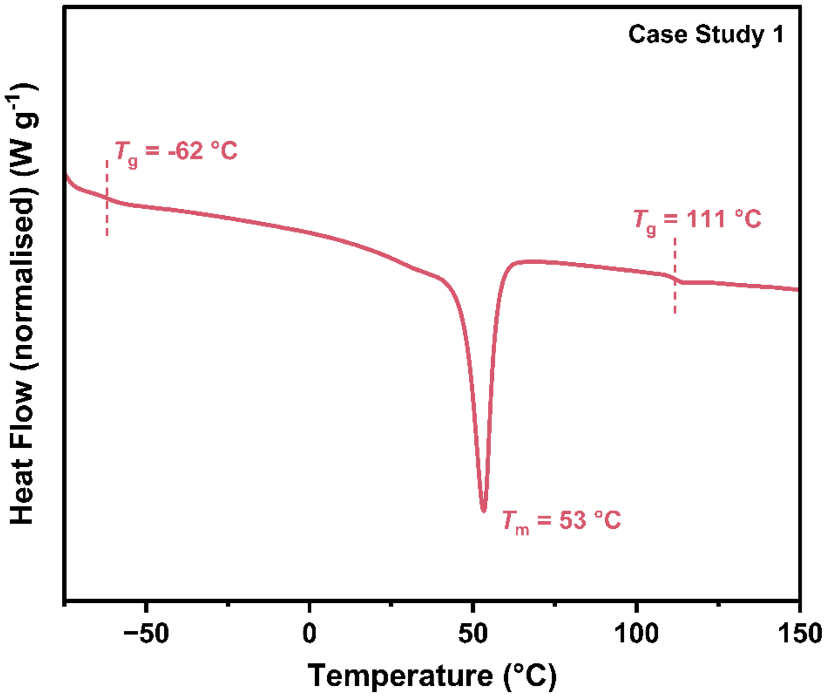


**Figure S7.** DSC trace (second heating curve) for case study 1.

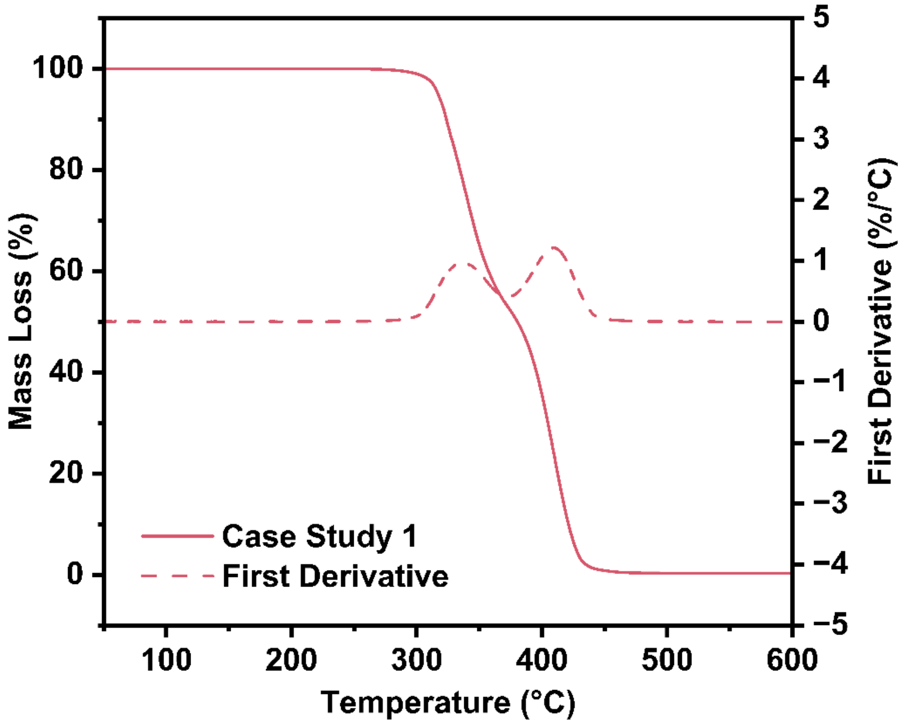


**Figure S8.** TGA thermogram for case study 1.

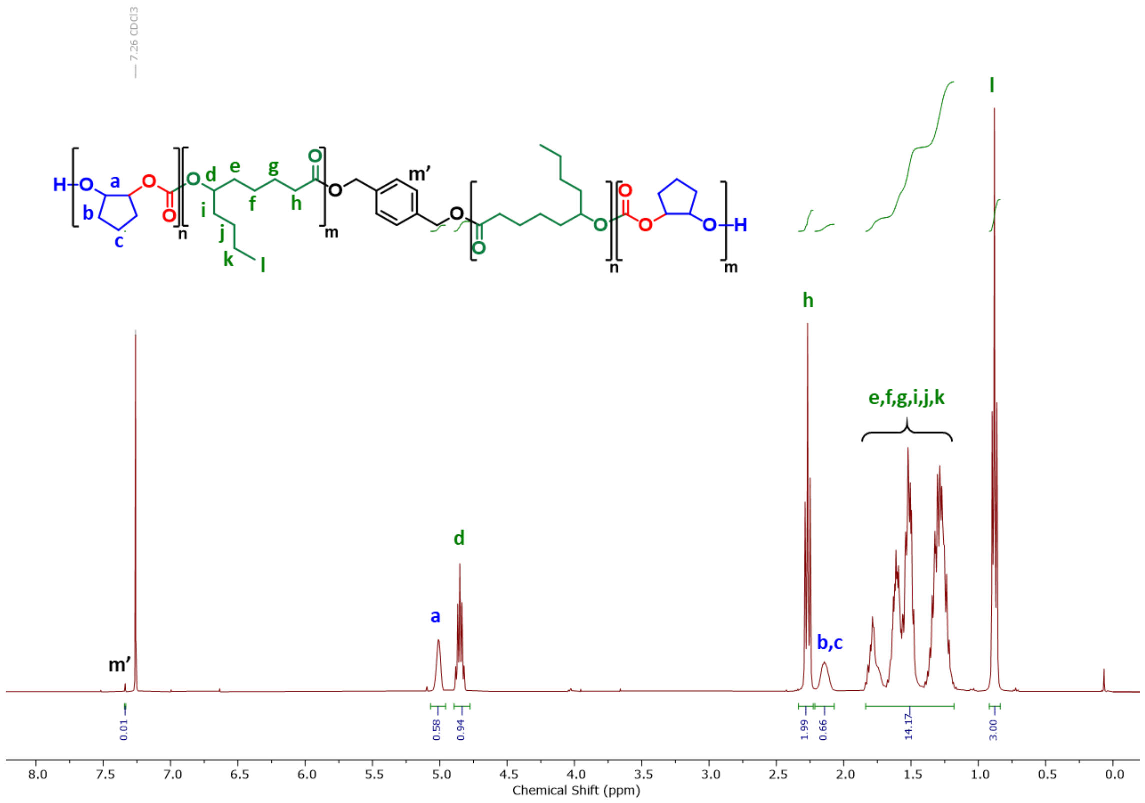


**Figure S9.** $^{1}H$ NMR (400 MHz, $CDCl_3$) spectrum of case study 2 poly(cyclopentene carbonate)-b-poly(ε-decalactone)-b-poly(cyclopentene carbonate).

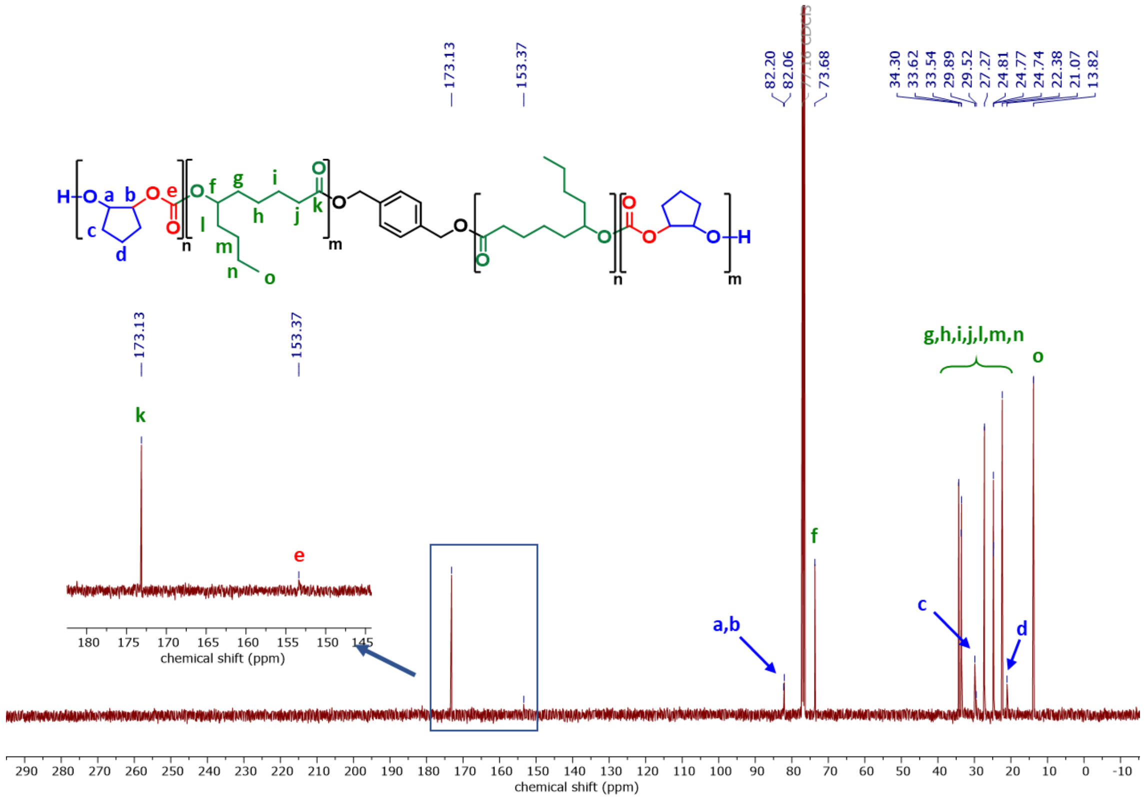


**Figure S10.** $^{13}C\{^{1}H\}$ NMR (400 MHz, $CDCl_3$) spectrum of case study 2.

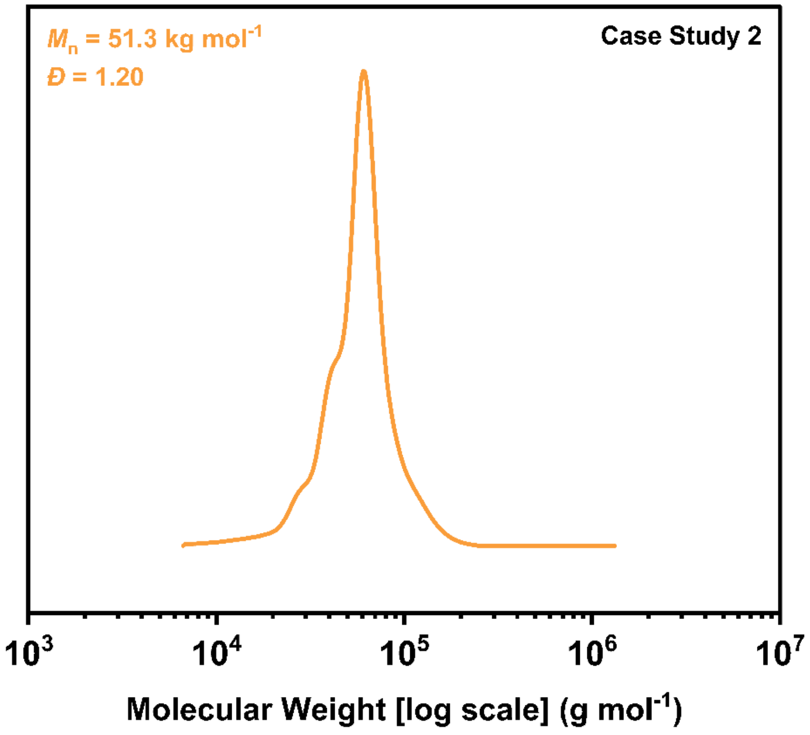


**Figure S11.** SEC trace (THF eluent, 1 mL min$^{-1}$) for case study 2.

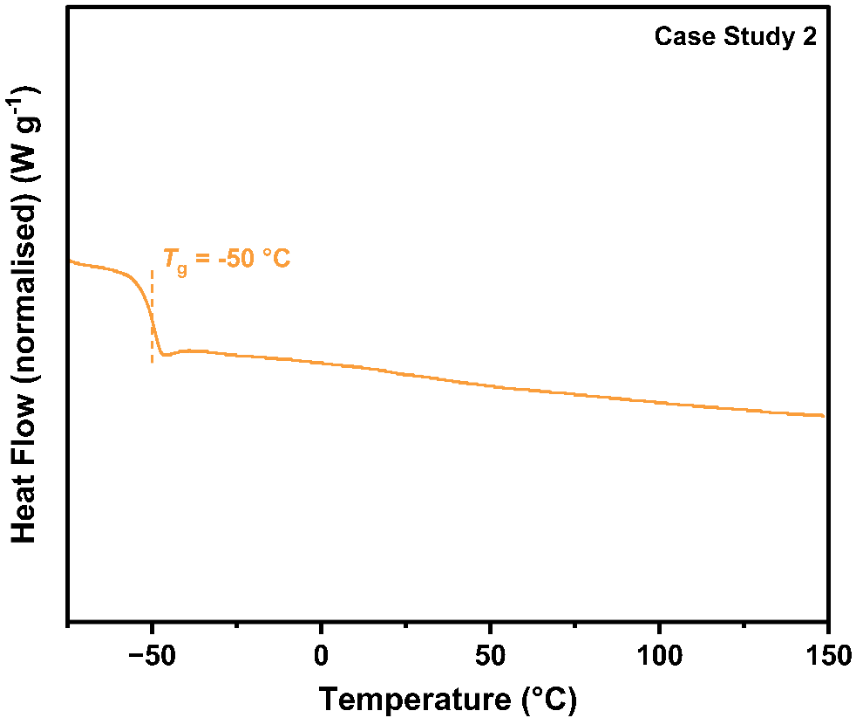


**Figure S12.** DSC trace (second heating curve) for case study 2.

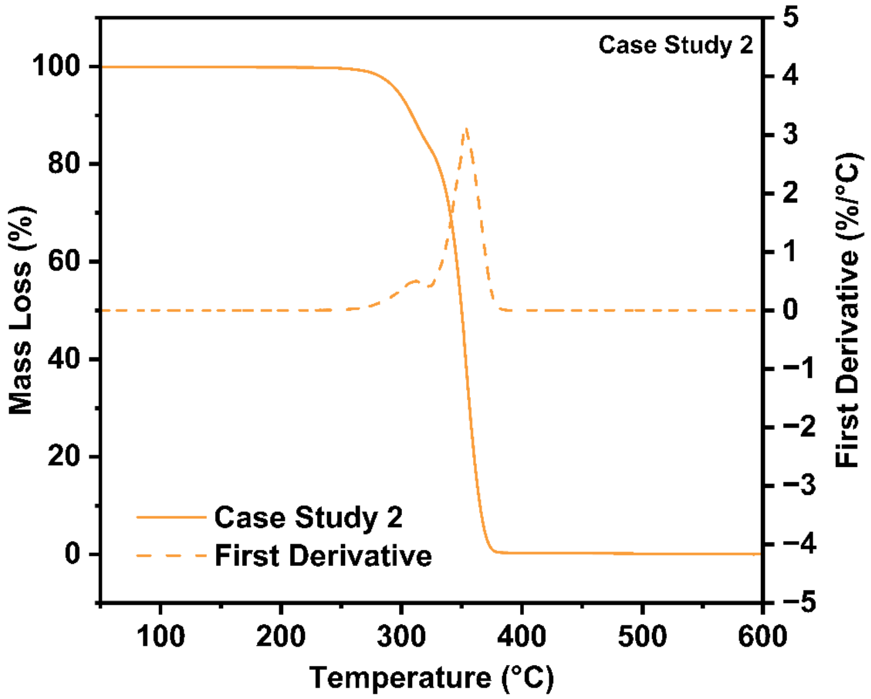


**Figure S13.** TGA thermogram for case study 2.

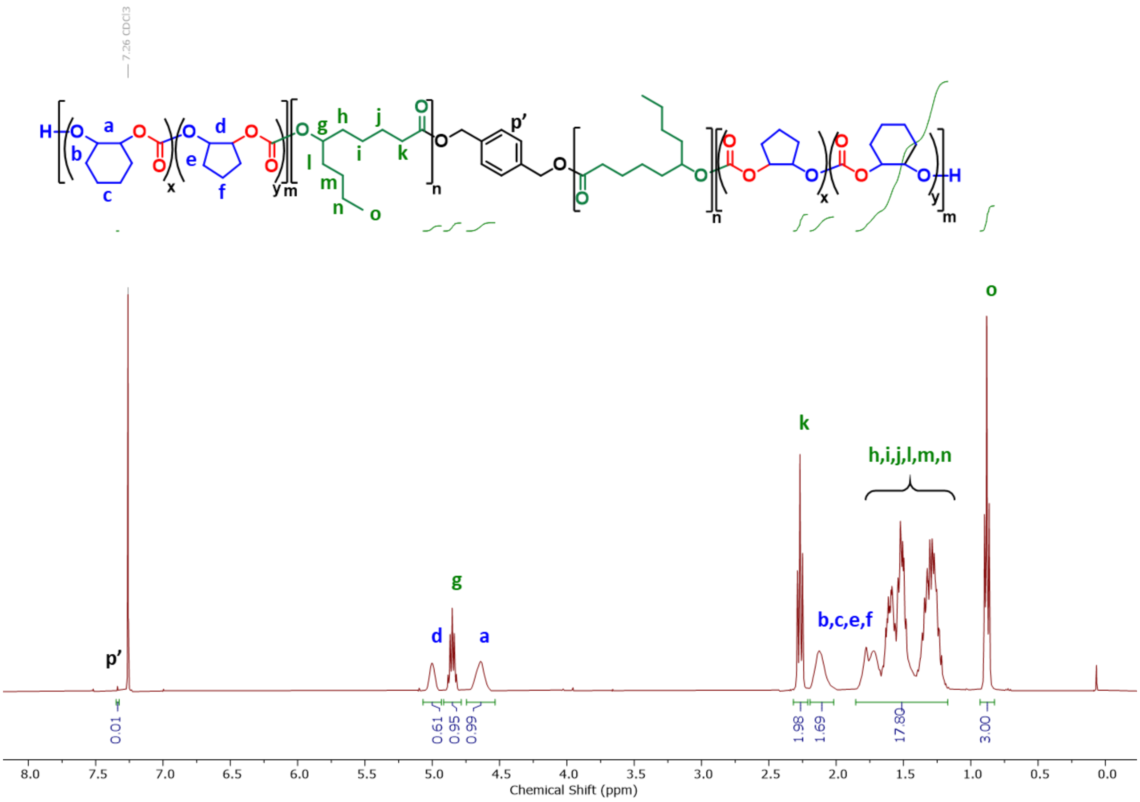

**Figure S14.** $^{1}H$ NMR (400 MHz, $CDCl_3$) spectrum of case study 3 poly(cyclohexene carbonate)-grad-poly(cyclopentene carbonate)-b-poly(ε-decalactone)-b-poly(cyclohexene carbonate)-grad-poly(cyclopentene carbonate).

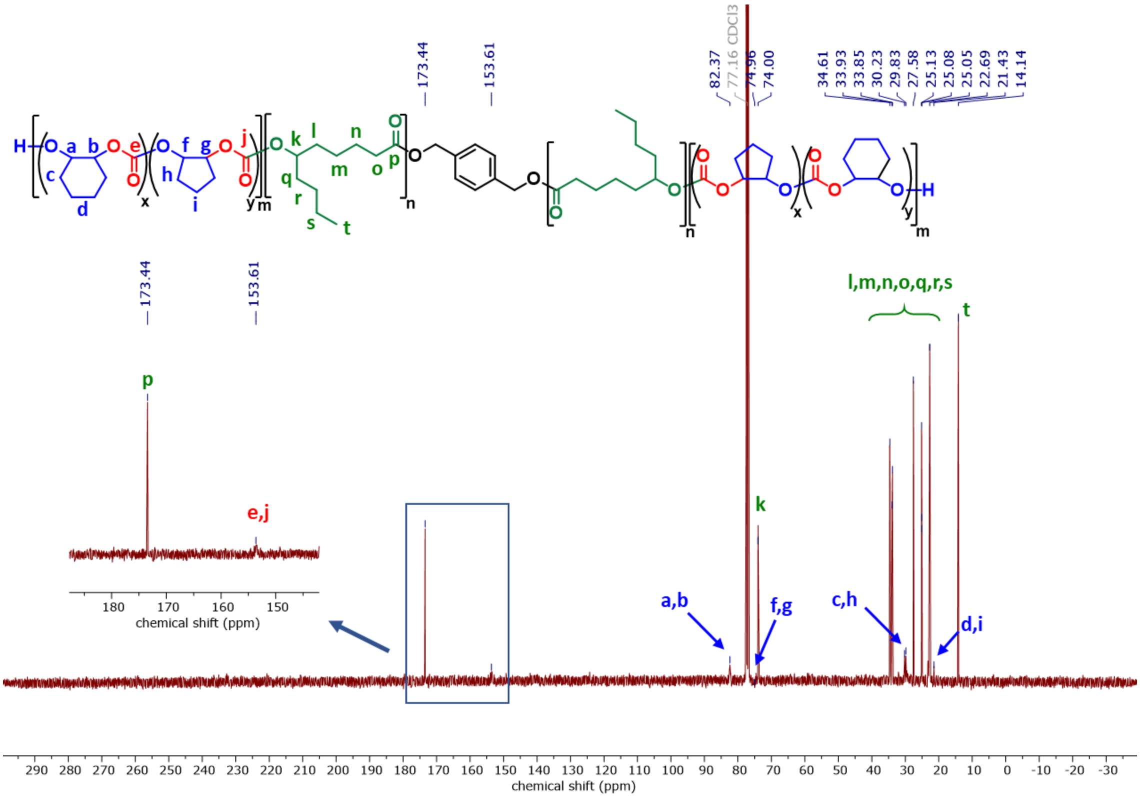

**Figure S15.** $^{13}C\{^{1}H\}$ NMR (400 MHz, $CDCl_3$) spectrum of case study 3.

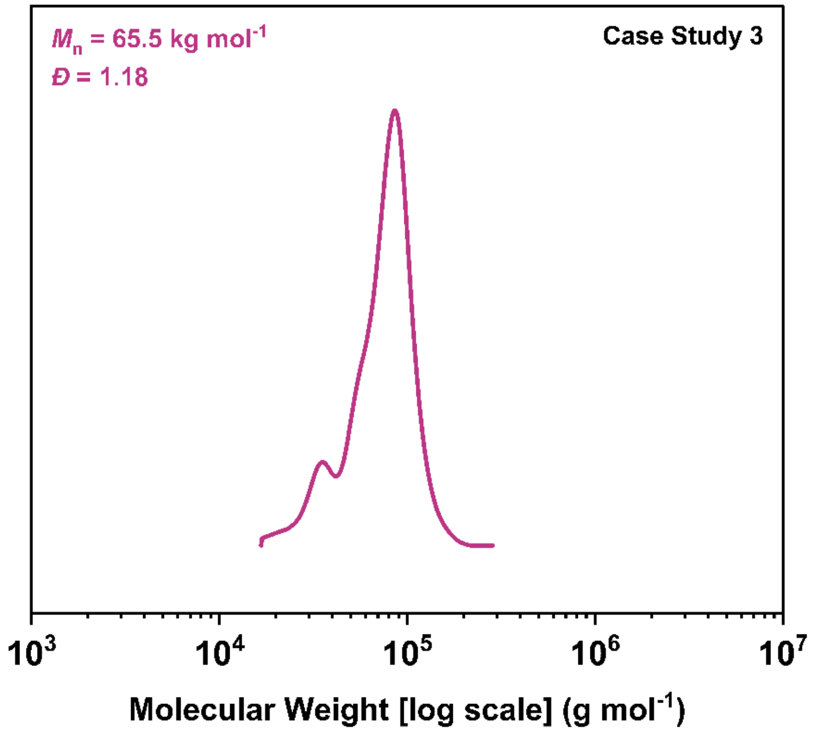


**Figure S16.** SEC trace (THF eluent, 1 mL min$^{-1}$) for case study 3.

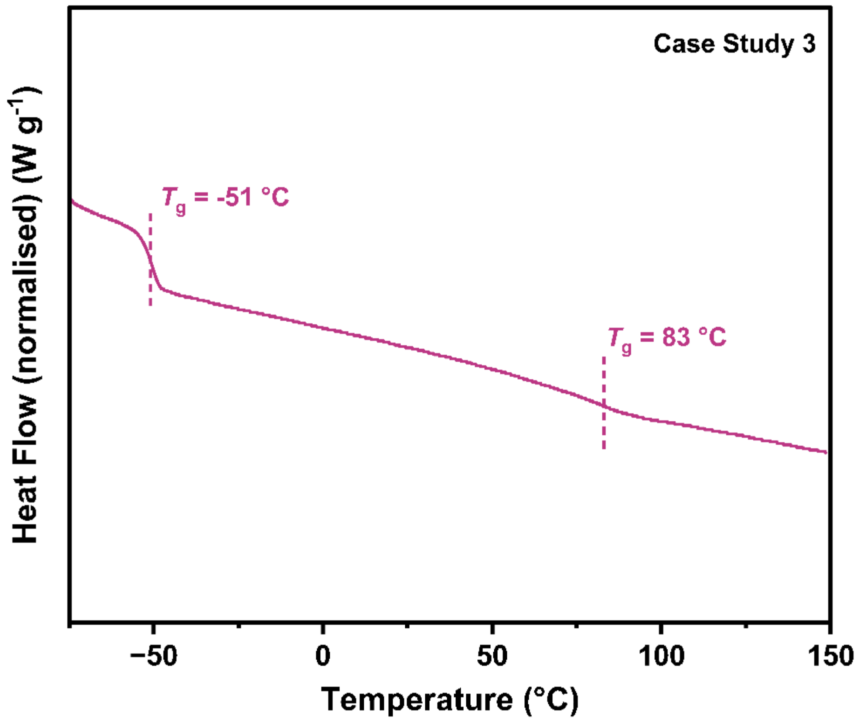


**Figure S17.** DSC trace (second heating curve) for case study 3.

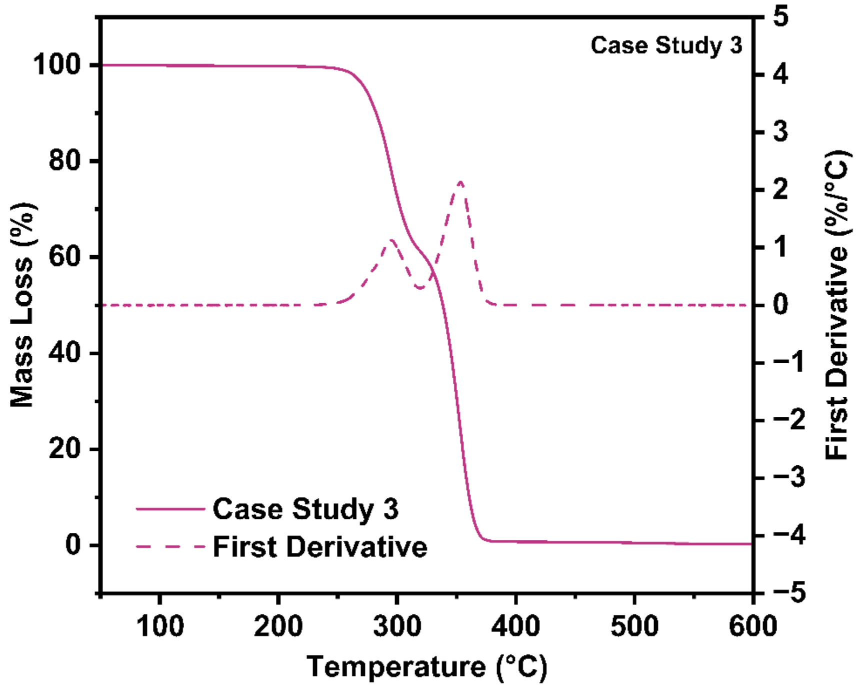


**Figure S18.** TGA thermogram for case study 3.

**Table S2.** Summary of Material Properties.

| **Sample** | $T_g$ (°C)[a] | $T_{d,5\%}$ (°C)[b] | $E_y$ (MPa)[c] | $\sigma$ (MPa)[d] | $\varepsilon_b$ (%)[e] | $U_T$ (MJ m$^{-3}$)[f] |
|---|---|---|---|---|---|---|
| **Case study 1** | -62, 111 | 317 | 515 ± 61 | 46.2 ± 6.4 | 1156 ± 101 | 311 ± 61 |
| **Case study 2** | -51 | 296 | 0.34 ± 0.08 | 0.34 ± 0.08 | 613 ± 78 | 1.15 ± 0.2 |
| **Case study 3** | -51, 83 | 273 | 82 ± 3 | 17.9 ± 1.0 | 1780 ± 64 | 157 ± 9 |

[a] Determined by DSC, second heating curve. [b] Temperature at 5% mass loss from TGA. [c] Young's modulus. [d] Tensile strength. [e] Strain at break. [f] Tensile toughness (area under the stress-strain curve). Mean values ± std. dev. from measurements conducted independently on five specimens.

## References


(1) Sulley, G. S.; Gregory, G. L.; Chen, T. T. D.; Peña Carrodeguas, L.; Trott, G.; Santmarti, A.; Lee, K.-Y.; Terrill, N. J.; Williams, C. K. Switchable Catalysis Improves the Properties of CO2-Derived Polymers: Poly(cyclohexene carbonate-b-ε-decalactone-b-cyclohexene carbonate) Adhesives, Elastomers, and Toughened Plastics. *J. Am. Chem. Soc.* **2020**, *142* (9), 4367–4378. DOI: 10.1021/jacs.9b13106.
